# DBU Promoted Polysubstituted Arene Formation via a Michael Addition/Cyclization/Elimination Cascade Reaction

**DOI:** 10.3390/molecules27238167

**Published:** 2022-11-23

**Authors:** Guishun Bai, Yang Yang, Xingyue Wang, Jiamin Wu, Hong Wang, Xinyi Ye, Xiaoze Bao

**Affiliations:** 1 College of Pharmaceutical Science & Collaborative Innovation Center of Yangtze River Delta Region Green Pharmaceuticals, Zhejiang University of Technology, Hangzhou 310014, China; 2 Zhejiang International Sci-Tech Cooperation Base for the Exploitation and Utilization of Nature Product, Hangzhou 310014, China; 3 Key Laboratory of Marine Fishery Resources Exploitment & Utilization of Zhejiang Province, Hangzhou 310014, China

**Keywords:** Michael addition, arenes, nitrostyrene, axial chirality

## Abstract

The straightforward construction of polysubstituted arenes is essential in both synthetic chemistry and medicinal chemistry. Herein, we reported a DBU promoted Michael addition/cyclization/elimination cascade reaction between vinylogous malononitrile derivatives and chlorinated nitrostyrenes for the synthesis of polysubstituted arenes. The method features mild reaction conditions, wide substrate scope and high yield. Interestingly, preliminary study of the enantioselective version of this cascade was conducted to give chiral biaryl atropisomers with up to 40% ee through center-to-axial chirality transfer strategy.

## 1. Introduction

The construction of arenes with different complexity is the main focus in both synthetic chemistry and medicinal chemistry [1,2,3,4], as the high frequency of the appearance of arenes in numerous valuable molecules [5,6,7,8]. Although lots of methodologies have been developed in this area, the facile synthesis of polysubstituted arenes usually relies on the inconvenient stepwise substitution of existed arenes [9,10,11,12]. Thus, the straightforward formation of arene cores with readily obtainable pre-functionalized substrates could enhance the synthetic efficiency and facilitate the development in this area.

**Figure 1:**
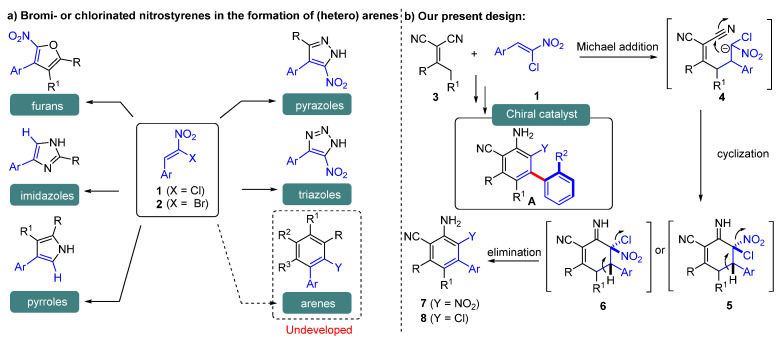
Recent development of bromi- or chlorinated nitrostyrenes in (hetero)arene synthesis.

Recently, the easily accessible α-brominated and chlorinated nitrostyrenes **1** and **2** [13,14] were emerged as powerful C_2_ synthons in the synthesis of different ring systems (Figure 1a). In particular, with the utilization of different C_3_ synthons, a series of polysubstituted heteroarenes were constructed through (3 + 2) cyclization process, including furans [15,16,17,18], pyrazoles [19,20,21], imidazoles [22], triazoles [23,24,25], and pyrroles [26,27,28,29,30]. The versatilities of nitrostyrenes **1-2** were based on the electron deficiency of the double bound and the high reactivity of the leaving group X (X= Cl, Br) [31,32,33]. Unfortunately, the construction of important polysubstituted arenes with these easily accessible nitrostyrenes were still undeveloped, probably due to the lack of suitable C_4_ synthons.

With the ongoing interest of our group in the chemistry of chlorinated nitrostyrene **1** [34,35], we hypothesized that the vinylogous malononitrile derivative **3** could undergo Michael addition with nitrostyrene **1** to form the adduct **4** in the presence of suitable base, then the base promoted intramolecular cyclization process could give the diastereomers **5** and **6** (Figure 1b). The final 1,2-trans elimination would furnish the polysubstituted arenes **7**-**8**, respectively. In this process, the diastereoselective control in the cyclization step was the key point to avoid the formation of the mixture of arenes **7** and **8**. Interestingly, with the chiral catalyst, the enantioselective Michael addition/cyclization/elimination cascade gave the opportunity to construct chiral biaryl atropisomers A trough center-to-axial chirality transfer process [36,37] (Figure 1b). Herein, we reported the preliminary results of this interesting cascade reaction.

## 2. Results and Discussion

To test our hypothesis, the initial reaction was conducted with 2-(3,4-dihydronaphthalen-1(2*H*)-ylidene) malononitrile **3a** with simple chlorinated nitrostyrene **1a** in the presence of stoichiometric amount of triethylamine (Et_3_N) in dichloromethane at room temperature (Table 1, entry 1). Unfortunately, no desired product **7aa** was detected, probably due to the weak basicity of Et_3_N. Next, 1,4-Diazabicyclo [2.2.2] octane (DABCO) instead of Et_3_N was used in the reaction, and the nitro group substituted product **7aa** was obtained in 29% yield in 2 h (Table 1, entry 2). With this promising result, 1,8-diazabicyclo [5.4.0] undec-7-ene (DBU) with enhancing basicity was tested to furnish **7aa** in 80% yield within 30 min, and no chlorinated arene derivative **8aa** was observed (Table 1, entry 3). However, when stronger base lithium *t*-butoxide was added into the reaction, only trace amount of **7aa** was observed (Table 1, entry 4). In order to further improve the yield, a series of inorganic bass were investigated, but very poor results were obtained (Table 1, entry 5–8). With DBU as the best choice of base, the solvent was then screened (Table 1, entry 9–12), and the utilization of toluene improved the yield of **7aa** to 85% within 15 min. In addition, enhancing the equivalent of DBU from 1.0 to 1.5 finally gave the desired product **7aa** in 95% yield.

**Scheme 1:**
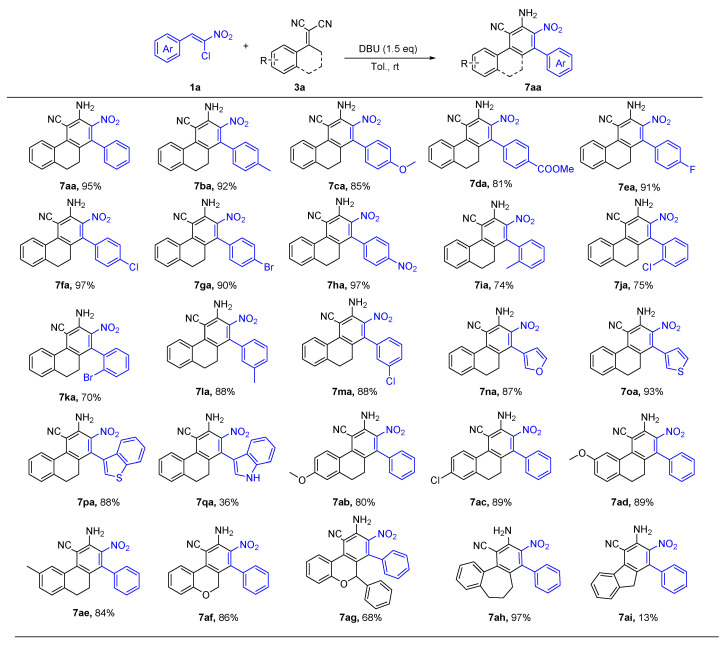
The scope of the substrates. **3** (0.2 mmol, 1.0 equiv), **1** (0.24 mmol, 1.2 equiv), and DBU (0.3 mmol, 1.5 equiv) were stirred at room temperature in toluene (2 mL).

With the optimized conditions in hand, the scope of vinylogous malononitrile derivative **3** and chlorinated nitrostyrenes **1** were explored. The results were summarized in Scheme 1. Both electron-withdrawing and electron-donating substituents in the phenyl ring of nitrostyrenes **1** were compatible in this reaction, and the resulting products were obtained in 70–97% yields (**7ba-7ma**). In general, the reaction proceeded better with electron-withdrawing groups compared with the electron-donating groups. Meanwhile, the yields of the products decreased in the order of *para*-substituent, *meta*-substituent, and *ortho*-substituent, probably due to the steric hindrance (**7ba**, **7la**, and **7ia**). Considering the importance of heterocyclic compounds in medicinal chemistry, the furan [38,39], thiophene [40,41,42,43], and indole [44,45,46,47] moieties were all installed to the arenes, and the desired products **7na-7qa** were furnished in 36–93% yields. On the other hand, the tolerance of vinylogous malononitrile derivative **3** were also investigated. Generally, the substituents on the phenyl ring had few influences in the yields of the products (**7ab-7ae**). The oxa-malononitrile derivatives were also suitable for this reaction, and the corresponding products **7af** and **7ag** were obtained in 86% and 68% yields, respectively. Interestingly, compared with the seven-membered ring fused malononitrile derivative, the five-membered ring fused one gave the product with much lower yields (**7ah** vs. **7ai**) with currently unknown reason.

**Scheme 2:**
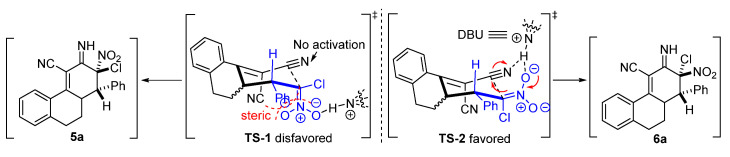
The proposed mechanism of the cyclization step. TS = transition state.

With these results in hand, the mechanism which was responsible for the dominant formation of the nitro-substituted arene **7** in this Michael addition/cyclization/elimination cascade was proposed (Scheme 2). After the formation of the first Michael adduct, two possible transition states **TS-1** and **TS-2** could be generated. In **TS-2**, the DBU spontaneously activated the α-position of nitro group and the electron-deficient nitrile group via the hydrogen bonding. In addition, the nitronate on the equatorial position may also possessed π-π stacking interaction with the nitrile group. Both of these could accelerate the cyclization process to form the key intermediate **6a**. Finally, the 1,2-trans elimination of HCl gave the observed product **7aa**. On the other hand, in **TS-2**, the hydrogen bonding network and the π-π stacking interaction were disrupted because of the distance between the nitronate and nitrile group. Moreover, the steric hindrance between the phenyl group and the nitronate also made this transition state unfavorable.

Recently, the enantioselective construction of axially chiral biaryls has attracted much attention, as their widely existence in natural products [48,49] and bioactive molecules [50,51,52], as well as in chiral ligands [53,54] and chiral organo-catalysts [55,56,57]. In this context, the successful synthesis of the *ortho*-brominated product **7ka** encouraged us to test the possibility of the enantioselective synthesis of this stable atropisomer. With the proposed mechanism and the currently emerged “center-to-axial chirality transfer” strategy, the chirality in the first Michael addition step could transfer to the final axial chirality. Next, the commonly used bifunctional chiral organo-catalysts were used to introduce the chirality (Table 2). Interestingly, with quinine **Cat. 1** as the catalyst, an intermediate was formed instead of the final product **7ka** (Table 2, entry 1). Unfortunately, the intermediate was not stable during the separation process. Benefit from the results in Table 1, the addition of DBU promoted the intermediate to the final product **7ka** in 70% yield and 17% ee. Considering the relatively weak basicity of quinine, the Michael adduct **9** was proposed to be the observed intermediate. This preliminary result promoted us to screen a series of cinchona alkaloid-derived catalysts to improve the enantioselectivity (Table 2, entries 2–8). The well-defined bifunctional thiourea/urea **Cat. 5-6** improved the enantioselectivities to 31% ee and 33% ee, respectively. Another bifunctional catalyst squaramide **Cat. 7** further improved the ee to 40% (more details could be found in the Supplementary Materials), albeit with lower 40% yield (Table 2, entries 7). Extending the carbon chain of the 3,5-bis(trifluoromethyl)aniline moiety did not give better result (Table 2, entries 8). In addition, the replacement of cinchona alkaloid moiety with chiral cyclohexane diamine also failed to improve the ee (Table 2, entries 9–10). Next, with **Cat. 7** as the catalyst, a quick survey of the solvent revealed that the ethyl acetate gave the best result, affording the product **7ka** in 63% yield and 40% ee (Table 2, entries 14). Rather than the high-throughput screening of the reaction conditions, the chirality conversion efficiency from the intermediate **9** to the final product **7ka** was essential for the enantioselectivity. Thus, in order to answer whether the relatively low ee was determined by the Michael addition step or by the chirality transfer step, the identification of the key intermediated **9** is still ongoing in our lab.

## 3. Materials and Methods

The detailed procedure of the synthesis and characterization of the products are given in Supplementary Materials.

## 4. Conclusions

In conclusion, a facile Michael addition/cyclization/elimination cascade reaction between vinylogous malononitrile derivatives and chlorinated nitrostyrenes was developed to construct polysubstituted arenes. The method features the advantages of mild conditions, wide substrate scope, and high yield. Preliminary studies based on center-to-axial chirality transfer strategy revealed that chiral biaryl atropisomers could be obtained through the enantioselective version of this cascade reaction. Further investigation on the key intermediate of this reaction and the improvement of the enantioselectivity are still in progress in our lab.

## Figures and Tables

**Table 1:**
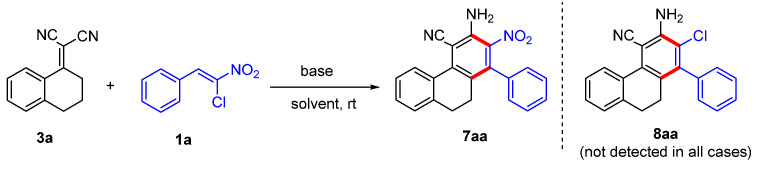
Optimization of the reaction conditions.

Entry ^a^	Solvent	Base	Reaction Time (h)	Yield ^b^ (%)
1 ^c^	DCM	Et_3_N	12	trace
2	DCM	DABCO	2	29
3	DCM	DBU	0.5	80
4	DCM	(CH_3_)_3_ COLi	12	trace
5	DCM	NaOH	12	trace
6	DCM	K_2_CO_3_	12	trace
7	DCM	CsCO_3_	12	16
8	DCM	K_3_PO_3_	12	trace
9 ^d^	EA	DBU	0.5	81
10	CH_3_CN	DBU	0.25	80
11 ^e^	DCE	DBU	0.25	82
12	Toluene	DBU	0.25	85
13 ^f^	Toluene	DBU	0.25	95

^a^ Reaction conditions: **1a** (0.12 mmol), **3a** (0.10 mmol), and base (0.10 mmol) were stirred in solvents (1.0 mL) at room temperature. ^b^ Isolated yield. ^c^ DCM = dichloromethane. ^d^ DCE = 1,2-dichloroethane. ^e^ EA = ethyl acetate. ^f^ DBU (0.15 mmol) was added.

**Table 2:**
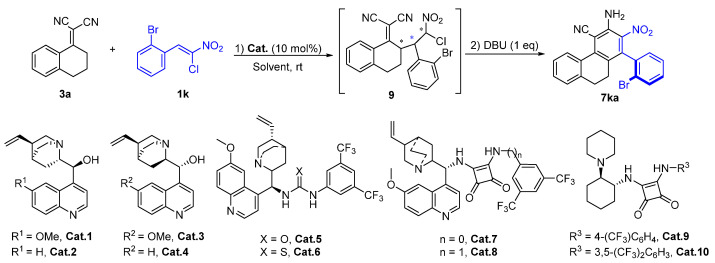
The optimization of the chiral product.

Entry ^a^	Cat.	Solvent	Yield (%)	Ee ^b^ (%)
1	**Cat. 1**	DCM	70	17
2	**Cat. 2**	DCM	63	−14
3	**Cat. 3**	DCM	71	23
4	**Cat. 4**	DCM	61	14
5	**Cat. 5**	DCM	65	31
6	**Cat. 6**	DCM	56	33
7	**Cat. 7**	DCM	40	40
8	**Cat. 8**	DCM	48	13
9	**Cat. 9**	DCM	48	−31
10	**Cat. 10**	DCM	67	−14
11	**Cat. 7**	DCE	38	37
12	**Cat. 7**	CHCl_3_	35	39
13	**Cat. 7**	Toluene	59	27
14	**Cat. 7**	EA	63	40

^a^ Reaction conditions: **1a** (0.10 mmol), **2q** (0.12 mmol), catalyst **Cat** (10 mol %) were stirred in solvent (1.0 mL) for 12 h at room temperature. Next, DBU (0.1 mmol) was added. ^b^ Ee values were determined by chiral HPLC analysis.

## Data Availability

The data presented in this study are available in the Supplementary Materials.

## Supplementary Materials

The following supporting information can be downloaded at: www.mdpi.com/article/10.3390/molecules27238167/s1, 1H and 13C NMR spectra for all compounds. References [13,58,59,60] are contained in the Supplementary Materials.
