# Supplementary material for: DBU Promoted Polysubstituted Arene Formation via a Michael Addition/Cyclization/Elimination Cascade Reaction
Source: Molecules. 2022 Nov 23;27(23):8167. doi: 10.3390/molecules27238167 (PMC9738611; doi:10.3390/molecules27238167)
Supplement: Supplementary file 1 [file molecules-27-08167-s001.zip › molecules-2034157-supplementary.pdf]

molecules

Supplementary Materials

DBU Promoted Polysubstituted Arene Formation via a Michael Addition/Cyclization/Elimination Cascade Reaction

Guishun Bai [1], Yang Yang [1], Xingyue Wang [1], Jiamin Wu [1], Hong Wang [1,2,3,*], Xinyi Ye [1,2,3,*] and Xiaoze Bao [1,2,3,*]

1 College of Pharmaceutical Science & Collaborative Innovation Center of Yangtze River Delta Region Green Pharmaceuticals, Zhejiang University of Technology, Hangzhou 310014, China; baiguishun@gmail.com (G.B.); 15550813128@163.com (Y.Y.); wangxingyue9@163.com (X.W.); WJM97830@163.com (J.W.)
2 Zhejiang International Sci-Tech Cooperation Base for the Exploitation and Utilization of Nature Product, Hangzhou 310014, China
3 Key Laboratory of Marine Fishery Resources Exploitment & Utilization of Zhejiang Province, Hangzhou 310014, China
* Correspondence: hongw@zjut.edu.cn (H.W.); xinyiye1020@zjut.edu.cn (X.Y.); baoxiaoze@zjut.edu.cn (X.B.)

Contents:

1. General information
2. Experimental procedures and characterization of products **7aa-7qa**, **7ab-7ai**
3. NMR spectra for compounds
4. Reference

1. General information

Unless otherwise noted, materials were purchased from commercial suppliers and used without further purification. Column chromatography was performed on silica gel (200~300 mesh). All ^{1}H NMR spectra were recorded on a Bruker AvanceII 500 MHzor Bruker AvanceII400 MHz respectively, ^{13}C NMR spectra were recorded on a Bruker AvanceII 101 MHz or Bruker Avance III 126 MHz with chemical shifts reported as ppm (in $CDCl_3$ or DMSO-d6., TMS as internal standard). Data for ^{1}H NMR are recorded as follows: chemical shift (δ, ppm), multiplicity (s = singlet, d = doublet, t = triplet, m = multiplet, br = broad singlet, dd = doublet doublet, coupling constants in Hz, integration). HRMS (ESI) was obtained with a HRMS/MS instrument (LTQ Orbitrap XL TM).

Vinylogous malononitrile derivative **3** were prepared according to the literature[1].Chloronitroalkenes **1** were prepared from varioussubstituted aldehyde according to the literature[2]. Catalysts **Cat.5-10** were synthesized according to literature procedures[3-4].

2. Experimental procedures and characterization of products

General procedure : synthesis of compound 7aa-7qa,7ab-7ai

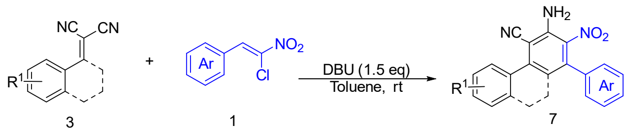

Vinylogous malononitrile derivative **3** (0.2 mmol), chloronitroolefins **1** (0.24 mmol, 1.2equiv) were added to a reaction tube, 2mL toluene were added and the mixture was stirred at rt. DBU (0.3mmol, 1.5 equiv) was added *via*a microsyringe, then the mixture was stirred at room temperature for 15 minutes. The reaction mixture was purified by column chromatography on silica gel directly to give the product **7**.

Enantioselective synthesis of compound 7ka

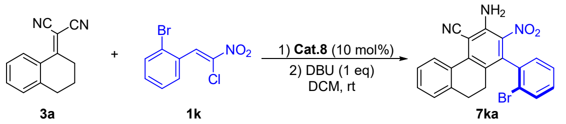

The mixture of α,α-dicyanoolefins **3a** (0.10 mmol), chloronitroolefins **1k** (0.12 mmol), and catalyst **Cat.7**(10 mol %) in DCM (1.0 mL) was stirred for 12 hours at room temperature. After then, DBU (0.1 mmol) was added and stirred for another 40 minutes.The reaction mixture was purified by column chromatography on silica gel directly to give the chiralproduct **7ka**.

4. -isocyano-2-nitro-1-phenyl-9,10-dihydrophenanthren-3-amine (7aa)

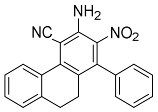

7aa

Prepared according to the general procedure as yellow solid (64 mg, 95% yield) after silica gel chromatography (petroleum ether/EtOAc = 10:1).^{1}H NMR (500 MHz, $CDCl_3$) δ 8.34-8.16 (m, 1H), 7.55-7.30 (m, 6H), 7.25-7.20 (m, 2H), 5.69 (s, 2H), 2.66 (dd, J = 8.3, 5.3 Hz, 2H), 2.47-2.34 (m, 2H). ^{13}C NMR (101 MHz, $CDCl_3$) δ 143.3, 142.6, 140.37, 139.81, 135.68, 131.13, 130.52, 128.81, 128.59, 128.19, 128.03 127.96, 127.45, 127.12, 117.24, 95.36, 29.06, 25.99. HRMS (ESI) m/z Calcd. for $C_{21}H_{16}N_3O_2^+$([M+H]$^+$) 342.1237, Found342.1244.

4. -isocyano-2-nitro-1-(p-tolyl)-9,10-dihydrophenanthren-3-amine (7ba)

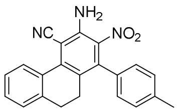

7ba

Prepared according to the general procedure as yellow solid (64 mg, 92% yield) after silica gel chromatography (petroleum ether/EtOAc = 10:1).^{1}H NMR (400 MHz, $CDCl_3$) δ 8.41-8.06 (m, 1H), 7.46-7.35 (m, 2H), 7.31-7.26 (m, 1H), 7.24 (s, 2H), 7.13-7.03 (m, 2H), 5.60 (s, 2H), 2.652.61 (m, 2H), 2.43-2.38(m, 2H), 2.40 (s, 3H).^{13}C NMR (101 MHz, $CDCl_3$) δ 143.12, 142.35, 140.38, 139.75, 138.52, 135.79, 132.54, 131.17, 130.40, 129.49, 128.31, 127.88, 127.39, 127.06, 117.24, 95.14, 29.05, 25.96, 21.35. HRMS (ESI) m/z Calcd. for $C_{22}H_{18}N_3O_2^+$ ([M+H]$^+$) 356.1394, Found 356.1395.

4. -isocyano-1-(4-methoxyphenyl)-2-nitro-9,10-dihydrophenanthren-3-amine (7ca)

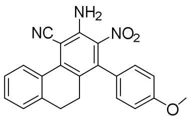

7ca

Prepared according to the general procedure as yellow solid (62 mg, 85% yield) after silica gel chromatography (petroleum ether/EtOAc = 10:1). 1H NMR (400 MHz, DMSO) δ 8.17-8.07 (m, 1H), 7.47-7.30 (m, 3H), 7.27-6.89 (m, 4H), 6.39 (s, 2H), 3.80 (s, 3H), 2.65-2.57 (m, 2H), 2.31-2.22 (m, 2H). ^{13}C NMR (101 MHz, DMSO) δ 159.81, 143.03, 141.06, 140.22, 138.59, 137.25, 131.40, 130.48, 130.09, 128.37, 127.55, 127.07, 126.98, 126.96 117.65, 114.53,

94.22, 55.62, 28.75, 25.98.HRMS (ESI) m/z Calcd. for$C_{22}H_{18}N_3O_3^+$([M+H]$^+$)372.1343, Found 372.1347.

methyl 4-(3-amino-4-isocyano-2-nitro-9,10-dihydrophenanthren-1-yl)benzoate (7da)

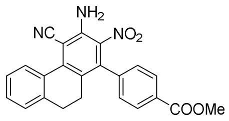

7da

Prepared according to the general procedure as yellow solid (64 mg, 81% yield) after silica gel chromatography (petroleum ether/EtOAc = 10:1).^{1}H NMR (400 MHz, $CDCl_3$) δ8.32-8.19 (m, 1H), 8.18-8.02 (m, 2H), 7.47-7.36 (m, 2H), 7.33-7.27 (m, 3H), 5.84 (s, 2H), 3.95 (s, 3H), 2.64 -2.63 (m, 2H), 2.44-2.23 (m, 2H).^{13}C NMR (101 MHz, $CDCl_3$) δ 166.52, 143.74, 143.10, 140.74, 139.77, 139.57, 134.65, 130.88, 130.75, 130.25, 130.07, 128.14, 127.99, 127.76, 127.52, 127.20, 117.03, 95.90, 52.36, 28.94, 25.96.HRMS (ESI) m/z Calcd. for$C_{23}H_{18}N_3O_4^+$([M+H]$^+$)400.1292,Found 400.1297.

1. -(4-fluorophenyl)-4-isocyano-2-nitro-9,10-dihydrophenanthren-3-amine (7ea)

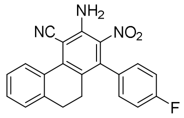

7ea

Prepared according to the general procedure as yellow solid (64 mg, 91% yield) after silica gel chromatography (petroleum ether/EtOAc = 10:1).^{1}H NMR (400 MHz, $CDCl_3$) δ 8.37-8.06 (m, 1H), 7.48-7.34 (m, 2H), 7.28-7.29 (m, 1H), 7.23-7.07 (m, 4H), 5.68 (s, 2H), 2.79-2.47 (m, 2H), 2.47-2.17 (m, 2H).^{13}C NMR (101 MHz, $CDCl_3$) δ 162.72 (d,*J* = 249.47 Hz), 143.31, 142.67, 139.69, 139.28, 131.50 (d, *J* = 3.7 Hz), 130.99, 130.59, 129.90, 129.82, 128.23, 127.94, 127.44, 127.15, 117.09, 115.99, (d, *J* = 22.2 Hz) 95.58, 28.98, 25.94.HRMS (ESI) m/z Calcd. for$C_{21}H_{15}FN_3O_2^+$([M+H]$^+$)360.1143, Found 360.1151.

1. -(4-chlorophenyl)-4-isocyano-2-nitro-9,10-dihydrophenanthren-3-amine (7fa)

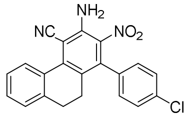

7fa

Prepared according to the general procedure as yellow solid (73 mg, 97% yield) after silica gel chromatography (petroleum ether/EtOAc = 10:1).^{1}H NMR (500 MHz, $CDCl_3$) δ 8.33-8.15 (m, 1H), 7.49-7.39 (m, 4H), 7.33-7.28 (m, 1H), 7.20-7.12 (m, 2H), 5.76 (s, 2H), 2.78-2.57 (m, 2H), 2.48-2.22 (m, 2H).^{13}C NMR (101 MHz, $CDCl_3$)δ 143.46, 142.83, 139.69, 139.16, 135.10, 134.69, 134.16, 130.92, 130.64, 129.38, 129.12, 128.01, 127.95, 127.45, 127.15, 117.04, 95.69, 28.93, 25.93.HRMS (ESI) m/z Calcd. for $C_{21}H_{15}ClN_3O_2^+$([M+H]$^+$)376.0847,Found 376.0854.

1. -(4-bromophenyl)-4-isocyano-2-nitro-9,10-dihydrophenanthren-3-amine (7ga)

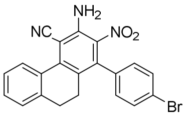

7ga

Prepared according to the general procedure as yellow solid (76 mg, 90% yield) after silica gel chromatography (petroleum ether/EtOAc = 10:1).^{1}H NMR (500 MHz, $CDCl_3$) δ 8.30-8.19 (m, 1H), 7.63-7.56 (m, 2H), 7.47-7.39 (m, 2H), 7.33-7.29 (m, 1H), 7.13-7.07 (m, 2H), 5.76 (s, 2H), 2.74-2.56 (m, 2H), 2.45-2.28 (m, 2H).^{13}C NMR (101 MHz, $CDCl_3$) δ 143.48, 139.69, 139.18, 134.68, 132.06, 130.91, 130.66, 129.64, 127.96, 127.64, 127.46, 127.16, 122.86, 117.03, 95.72, 28.93, 25.94.HRMS (ESI) m/z Calcd. for $C_{21}H_{15}BrN_3O_2^+$([M+H]$^+$)420.0342,Found 420.0352.

4. -isocyano-2-nitro-1-(4-nitrophenyl)-9,10-dihydrophenanthren-3-amine (7ha)

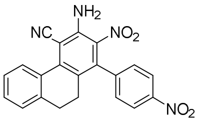

7ha

Prepared according to the general procedure as yellow solid (74 mg, 97% yield) after silica gel chromatography (petroleum ether/EtOAc = 10:1).^{1}H NMR (500 MHz, $CDCl_3$) δ 8.37-8.31 (m, 2H), 8.29-8.23 (m, 1H), 7.49-7.44 (m, 2H), 7.44-7.40 (m, 2H), 7.34-7.29 (m, 1H), 6.04 (s, 2H), 2.76-2.60 (m, 2H), 2.41-2.24 (m, 2H).^{13}C NMR (101 MHz, $CDCl_3$) δ 147.74, 144.16, 143.68, 143.15, 139.68, 138.41, 133.86, 131.03, 130.61, 129.08, 128.06, 127.60, 127.44, 127.31, 124.10, 116.79, 96.52, 28.82, 26.00.HRMS (ESI) m/z Calcd. for $C_{21}H_{15}N_4O_4^+$([M+H]$^+$)387.1088,Found 387.1094.

4. -isocyano-2-nitro-1-(o-tolyl)-9,10-dihydrophenanthren-3-amine (7ia)

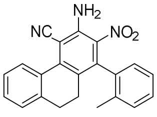

7ia

Prepared according to the general procedure as yellow solid (53 mg, 74% yield) after silica gel chromatography (petroleum ether/EtOAc = 10:1).^{1}H NMR (400 MHz, $CDCl_3$) δ 8.28 (dd, *J* = 7.4, 1.8 Hz, 1H), 7.41 (tt, *J* = 7.5, 5.9 Hz, 2H), 7.33-7.25 (m, 3H), 7.237.20 (m, 1H), 7.00 (d, *J*= 7.5 Hz, 1H), 5.72 (s, 2H), 2.65 (t, *J* = 6.8 Hz, 2H), 2.25 (td, *J* = 6.4, 3.3 Hz, 2H), 2.13 (s, 3H).^{13}C NMR (101 MHz, $CDCl_3$) δ 143.59, 142.25, 140.16, 139.90, 136.31, 135.33, 131.03, 130.48, 130.14, 128.63, 128.13, 127.97, 127.43, 127.31, 127.10, 126.14, 117.22, 95.29, 28.97, 25.19, 19.69.HRMS (ESI) m/z Calcd. for$C_{22}H_{18}N_3O_2^+$([M+H]$^+$)356.1394,Found 356.1400.

1. -(2-chlorophenyl)-4-isocyano-2-nitro-9,10-dihydrophenanthren-3-amine (7ja)

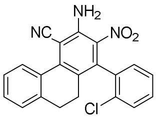

7ja

Prepared according to the general procedure as yellow solid (56 mg, 75% yield) after silica gel chromatography (petroleum ether/EtOAc = 10:1).^{1}H NMR (400 MHz, $CDCl_3$) δ 8.54-8.04 (m, 1H), 7.62-7.46 (m, 1H), 7.46-7.26 (m, 5H), 7.13 (dd, *J* = 7.4, 2.0 Hz, 1H), 5.99 (s, 2H), 2.79-2.57 (m, 2H), 2.47-2.12 (m, 2H).^{13}C NMR (101 MHz, $CDCl_3$) δ 144.20, 140.05, 133.25, 130.86, 130.69, 129.84, 129.69, 129.03, 128.39, 127.99, 127.54, 127.19, 127.12, 117.11, 28.87, 25.36.HRMS (ESI) m/z Calcd. for$C_{21}H_{15}ClN_3O_2^+$([M+H]$^+$)376.0847,Found 376.0855.

1. -(2-bromophenyl)-4-isocyano-2-nitro-9,10-dihydrophenanthren-3-amine (7ka)

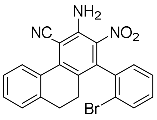

7ka

Prepared according to the general procedure as yellow solid (60 mg, 70% yield) after silica gel chromatography (petroleum ether/EtOAc = 10:1).^{1}H NMR (500 MHz, $CDCl_3$) δ 8.34-8.27 (m, 1H), 7.71 (dd, *J* = 8.0, 1.2 Hz, 1H), 7.50-7.36 (m, 3H), 7.35-7.28 (m, 2H), 7.15 (dd, *J* = 7.6, 1.7 Hz, 1H), 6.03 (s, 2H), 2.71 (qdd, *J* = 14.7, 8.6, 5.0 Hz, 2H), 2.47-2.14 (m, 2H).^{13}C NMR (126 MHz, $CDCl_3$) δ 144.26, 143.08, 140.10, 139.49, 137.40, 134.14, 132.84, 130.89, 130.71, 129.89, 129.13, 128.24, 128.00, 127.75, 127.54, 127.13, 123.19, 117.12, 96.11, 28.91, 25.48.HRMS (ESI) m/z Calcd. for$C_{21}H_{15}BrN_3O_2^+$([M+H]$^+$)420.0342,Found 420.0349.Enantiomeric excess was determined to be 40% (determined by HPLC using chiral IC column, hexane/2-propanol = 7/3, λ = 254 nm, 1.0 mL/min, t_{maJor} = 29.4 min, t_{minor} = 33.7 min).

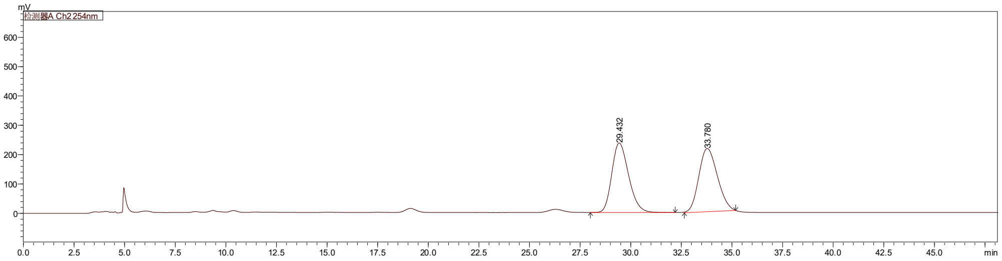

Detector A 254nm

峰号	保留时间	面积	高度	标记	浓度	浓度单位	化合物ID号	化合物名	面积%
1	29.432	13756612	237204		49.926				49.926
2	33.780	13797213	216014	M	50.074				50.074
总计		27553826	453218		100.000				100.000

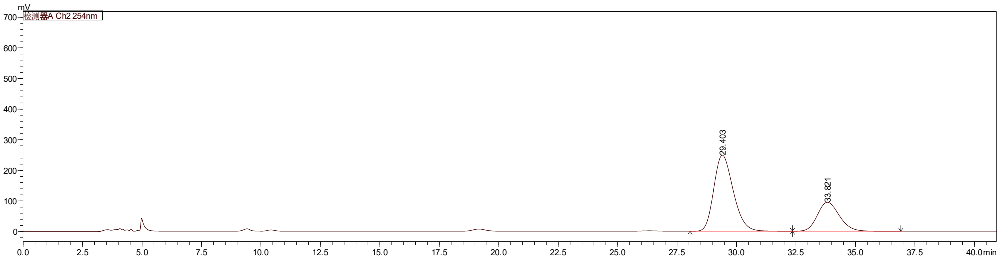

Detector A 254nm

峰号	保留时间	面积	高度	标记	浓度	浓度单位	化合物ID号	化合物名	面积%
1	29.402	38694715	680368	M	69.941				69.941
2	33.820	16630336	260344	M	30.059				30.059
总计		55325051	940712		100.000				100.000

4. -isocyano-2-nitro-1-(m-tolyl)-9,10-dihydrophenanthren-3-amine (7ia)

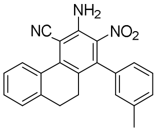

7Ia

Prepared according to the general procedure as yellow solid (63 mg, 88% yield) after silica gel chromatography (petroleum ether/EtOAc = 10:1).^{1}H NMR (400 MHz, $CDCl_3$) δ 8.33-8.06 (m, 1H), 7.48-7.36 (m, 2H), 7.35-7.26 (m, 2H), 7.257.15 (m, 1H), 7.06-6.92 (m, 2H), 5.63 (s, 2H), 2.72-2.54 (m, 2H), 2.44-2.39 (m, 2H), 2.38 (s, 3H).^{13}C NMR (101 MHz, $CDCl_3$) δ 143.18, 142.41, 140.51, 139.78, 138.50, 135.58, 135.51, 131.14, 130.42, 129.33, 128.62, 128.45, 128.22, 127.89, 127.39, 127.06, 125.14, 117.23, 95.17, 29.05, 25.97, 21.48.HRMS (ESI) m/z Calcd. for$C_{22}H_{18}N_3O_2^+$([M+H]+)356.1394,Found 356.1401.

1. -(3-chlorophenyl)-4-isocyano-2-nitro-9,10-dihydrophenanthren-3-amine (7ma)

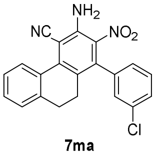

7ma

Prepared according to the general procedure as yellow solid (66 mg, 88% yield) after silica gel chromatography (petroleum ether/EtOAc = 10:1).^{1}H NMR (400 MHz, $CDCl_3$) δ 8.29-8.17 (m, 1H), 7.47-7.34 (m, 4H), 7.32-7.26 (m, 1H), 7.23-7.16 (m, 1H), 7.09 (dt, *J* = 6.5, 1.9 Hz, 1H), 5.79 (s, 2H), 2.73-2.59 (m, 2H), 2.45-2.26 (m, 2H).^{13}C NMR (101 MHz, $CDCl_3$) δ143.58, 139.73, 138.90, 137.55, 134.82, 134.75, 130.87, 130.69, 130.10, 128.73, 128.06, 127.98, 127.96, 127.47, 127.16, 126.23, 117.01, 28.94, 25.95.HRMS (ESI) m/z Calcd. For $C_{21}H_{15}ClN_3O_2^+$([M+H]+) 376.0847,Found 376.0856.

1. -(furan-3-yl)-4-isocyano-2-nitro-9,10-dihydrophenanthren-3-amine (7na)

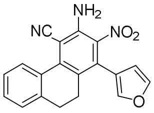

7na

Prepared according to the general procedure as yellow solid (57 mg, 87% yield) after silica gel chromatography (petroleum ether/EtOAc = 10:1).^{1}H NMR (400 MHz, $CDCl_3$) δ 8.25-8.05 (m, 1H), 7.54 (d, *J* = 1.8 Hz, 1H), 7.46-7.37 (m, 2H), 7.35-7.27 (m, 1H), 6.61-6.45 (m, 2H), 5.77 (s, 2H), 2.70 (s, 4H).^{13}C NMR (101 MHz, DMSO) δ 146.13, 145.11, 143.61, 142.38, 140.21, 135.11, 131.17, 130.73, 128.31, 127.95, 127.66, 127.09, 126.90, 117.43, 113.62, 112.29, 95.90, 28.68, 25.67.HRMS (ESI) m/z Calcd. for $C_{19}H_{14}N_3O_3^+$([M+H]+)332.1030,Found 332.1037.

4. -isocyano-2-nitro-1-(thiophen-3-yl)-9,10-dihydrophenanthren-3-amine (7oa)

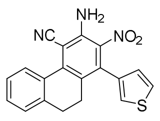

7oa

Prepared according to the general procedure as yellow solid (65 mg, 93% yield) after silica gel chromatography (petroleum ether/EtOAc = 10:1).^{1}H NMR (400 MHz, $CDCl_3$) δ 8.41-7.96 (m, 1H), 7.54-7.44 (m, 1H), 7.44-7.35 (m, 2H), 7.33-7.27 (m, 1H), 7.147.05 (m, 1H), 7.016.92 (m, 1H), 5.58 (s, 2H), 2.78-2.45 (m, 4H).^{13}C NMR (101 MHz, $CDCl_3$) δ 142.93, 142.32, 139.70, 136.24, 134.86, 132.88, 130.94, 130.56, 129.74, 128.13, 127.94, 127.67, 127.40, 127.12, 117.00, 96.12, 28.96, 26.03.HRMS (ESI) m/z Calcd. for $C_{19}H_{14}N_3O_2S^+$([M+H]$^+$)348.0801,Found 348.0805.

1. -(benzo[b]thiophen-3-yl)-4-isocyano-2-nitro-9,10-dihydrophenanthren-3-amine (7pa)

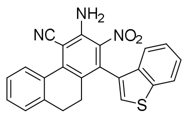

7pa

Prepared according to the general procedure as yellow solid (70 mg, 88% yield) after silica gel chromatography (petroleum ether/EtOAc = 10:1).^{1}H NMR (500 MHz, $CDCl_3$) δ 8.47 (s, 1H), 8.28 (dd, J = 7.6, 1.5 Hz, 1H), 7.48-7.38 (m, 3H), 7.34-7.28 (m, 2H), 7.20 (d, J = 2.6 Hz, 1H), 7.15 (ddd, J = 8.0, 7.1, 1.0 Hz, 1H), 5.46 (s, 2H), 2.68-2.57 (m, 3H), 2.512.41 (m, 1H).^{13}C NMR (101 MHz, $CDCl_3$) δ 143.57, 139.89, 130.92, 130.87, 130.62, 129.39, 128.00, 127.46, 127.16, 125.01, 124.83, 124.47, 124.44, 122.96, 122.30, 117.11, 28.97, 25.67.HRMS (ESI) m/z Calcd. for $C_{23}H_{16}N_3O_2S^+$([M+H]$^+$)398.0958,Found 398.0963.

1. -(1H-indol-3-yl)-4-isocyano-2-nitro-9,10-dihydrophenanthren-3-amine (7qa)

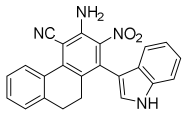

7qa

Prepared according to the general procedure as yellow solid (36 mg, 27% yield) after silica gel chromatography (petroleum ether/EtOAc = 10:1).^{1}H NMR (500 MHz, $CDCl_3$) δ 8.47 (s, 1H), 8.28 (dd, *J* = 7.6, 1.5 Hz, 1H), 7.48-7.38 (m, 3H), 7.34-7.28 (m, 2H), 7.277.25 (m, 1H), 7.20 (d, *J* = 2.6 Hz, 1H), 7.15 (ddd, *J* = 8.0, 7.1, 1.0 Hz, 1H), 5.46 (s, 2H), 2.68-2.57 (m, 3H), 2.51-2.43 (m, 1H).^{13}C NMR (101 MHz, $CDCl_3$) δ 142.69, 141.74, 139.85, 135.79, 133.24, 131.40, 130.23, 129.96, 127.86, 127.34, 127.03, 126.63, 123.23, 122.99, 120.75, 119.43, 117.43, 111.65, 110.72, 94.88, 29.16, 26.03.HRMS (ESI) m/z Calcd. for $C_{23}H_{17}N_4O_2^+$([M+H]$^+$)381.1346,Found 381.1352.

4. -isocyano-7-methoxy-2-nitro-1-phenyl-9,10-dihydrophenanthren-3-amine (7ab)

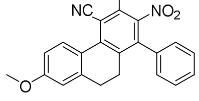

7ab

Prepared according to the general procedure as yellow solid (58 mg, 80% yield) after silica gel chromatography (petroleum ether/EtOAc = 10:1).^{1}H NMR (500 MHz, $CDCl_3$) δ 8.22 (d, *J* = 8.8 Hz, 1H), 7.50-7.39 (m, 3H), 7.24-7.18 (m, 2H), 6.96 (dd, *J* = 8.7, 2.7 Hz, 1H), 6.81 (d, *J* = 2.7 Hz, 1H), 5.73 (s, 2H), 3.88 (s, 3H), 2.71-2.55 (m, 2H), 2.46-2.30 (m, 2H).^{13}C NMR (101 MHz, $CDCl_3$) δ 161.28, 143.49, 142.78, 141.98, 140.32, 135.94, 134.68, 129.13, 128.72, 128.40, 127.96, 127.29, 123.87, 117.49, 113.64, 112.04, 94.48, 55.42, 29.43, 25.89.HRMS (ESI) m/z Calcd. for$C_{22}H_{18}N_3O_3^+$([M+H]$^+$)372.1343,Found 372.1347.

7. -chloro-4-isocyano-2-nitro-1-phenyl-9,10-dihydrophenanthren-3-amine (7ac)

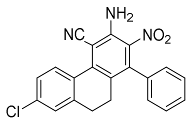

7ac

Prepared according to the general procedure as yellow solid (66 mg, 89% yield) after silica gel chromatography (petroleum ether/EtOAc = 10:1).^{1}H NMR (400 MHz, $CDCl_3$) δ 8.17 (d, *J* = 8.5 Hz, 1H), 7.52-7.41 (m, 3H), 7.39 (dd, J = 8.5, 2.3 Hz, 1H), 7.28 (d, *J* = 2.2 Hz, 1H), 7.24-7.11 (m, 2H), 5.64 (s, 2H), 2.61 (dd, *J* = 8.4, 5.3 Hz, 2H), 2.43-2.30 (m, 2H).^{13}C NMR (101 MHz, $CDCl_3$) δ143.26, 141.60, 141.37, 140.47, 136.29, 135.69, 135.39, 129.59, 128.83, 128.67, 128.64, 128.02, 127.94, 127.81, 127.24, 116.98, 95.16, 28.92, 25.73.HRMS (ESI) m/z Calcd. for $C_{21}H_{15}ClN_3O_2^+$([M+H]$^+$)376.0847,Found 376.0855.

4. -isocyano-6-methoxy-2-nitro-1-phenyl-9,10-dihydrophenanthren-3-amine (7ad)

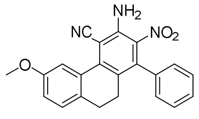

7ad

Prepared according to the general procedure as yellow solid (66 mg, 89% yield) after silica gel chromatography (petroleum ether/EtOAc = 10:1).^{1}H NMR (500 MHz, $CDCl_3$) δ 7.82 (d, *J* = 2.5 Hz, 1H), 7.49-7.40 (m, 3H), 7.25-7.17 (m, 3H), 6.97 (dd, *J*= 8.3, 2.6 Hz, 1H), 5.67 (s, 2H), 3.93 (s, 3H), 2.65-2.51 (m, 2H), 2.47-2.30 (m, 2H).^{13}C NMR (101 MHz, $CDCl_3$) δ 158.43, 143.20, 142.52, 140.32, 135.64, 131.84, 131.77, 128.76, 128.54, 128.32, 127.99, 117.23, 117.01, 112.20, 95.30, 55.64, 28.05, 26.28.HRMS (ESI) m/z Calcd. for $C_{22}H_{18}N_3O_3^+$([M+H]$^+$)372.1343,Found 372.1347.

4. -isocyano-6-methyl-2-nitro-1-phenyl-9,10-dihydrophenanthren-3-amine (7ae)

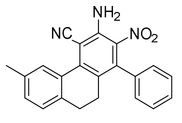

7ae

Prepared according to the general procedure as yellow solid (64 mg, 95% yield) after silica gel chromatography (petroleum ether/EtOAc = 10:1). ^{1}H NMR (500 MHz, $CDCl_3$) δ 8.14-8.02 (m, 1H), 7.50-7.41 (m, 3H), 7.26-7.16 (m, 4H), 5.70 (s, 2H), 2.65-2.57 (m, 2H), 2.48 (s, 3H), 2.42-2.35 (m, 2H). ^{13}C NMR (101 MHz, $CDCl_3$) δ 143.32, 142.73, 140.32, 136.81, 136.74, 135.77, 135.43, 131.23, 131.00, 128.80, 128.55, 128.25, 128.03, 127.80, 117.26, 95.30, 28.60, 26.23, 21.35.HRMS (ESI) m/z Calcd. for $C_{22}H_{18}N_3O_2^+$([M+H]$^+$)356.1394,Found 356.1398.

10. -isocyano-8-nitro-7-phenyl-6H-benzo[c]chromen-9-amine (7af)

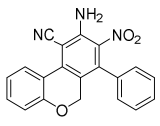

7af

Prepared according to the general procedure as yellow solid (59 mg, 86% yield) after silica gel chromatography (petroleum ether/EtOAc = 10:1).^{1}H NMR (500 MHz, $CDCl_3$) δ 8.41 (dd, J = 8.0, 1.5 Hz, 1H), 7.55-7.37 (m, 4H), 7.24-7.13 (m, 3H), 7.05 (dd, J = 8.2, 1.2 Hz, 1H), 5.86 (s, 2H), 4.63 (s, 2H).^{13}C NMR (101 MHz, $CDCl_3$) δ 156.71, 144.61, 139.03, 137.50, 134.76, 134.01, 132.87, 129.09, 129.00, 127.64, 126.58, 122.66, 122.58, 120.32, 117.85, 116.65, 93.71, 65.59.HRMS (ESI) m/z Calcd. for$C_{20}H_{14}N_3O_3^+$([M+H]$^+$)344.1030,Found 344.1038.

10. -isocyano-8-nitro-6,7-diphenyl-6H-benzo[c]chromen-9-amine (7ag)

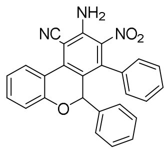

7ag

Prepared according to the general procedure as yellow solid (57 mg,68% yield) after silica gel chromatography (petroleum ether/EtOAc = 10:1).^{1}H NMR (500 MHz, $CDCl_3$) δ 8.42 (dd, J = 8.0, 1.5 Hz, 1H), 7.44 (tdd,*J* = 7.7, 1.5, 0.6 Hz, 1H), 7.36 (tt, *J* = 7.5, 1.3 Hz, 1H), 7.23-7.10 (m, 4H), 7.07 (ddd, *J* = 8.1, 7.4, 1.2 Hz, 1H), 7.04-6.96 (m, 2H), 6.86 (dd, *J* = 8.1, 1.2 Hz, 1H), 6.80-6.73 (m, 1H), 5.88 (s, 2H), 5.83 (s, 1H).^{13}C NMR (101 MHz, $CDCl_3$) δ 153.91, 144.80, 137.87, 133.59, 132.97, 128.94, 128.80, 128.61, 128.55, 128.20, 127.13, 126.03, 123.45, 122.40, 119.08, 116.76, 75.18.HRMS (ESI) m/z Calcd. for $C_{26}H_{18}N_3O_3^+$([M+H]$^+$)420.1343,Found 420.1350.

2. -amino-3-nitro-4-phenyl-6,7-dihydro-5H-dibenzo[a,c][7]annulene-1-carbonitrile (7ah)

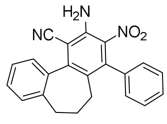

7ah

Prepared according to the general procedure as yellow solid (70 mg, 97% yield) after silica gel chromatography (petroleum ether/EtOAc = 10:1).^{1}H NMR (500 MHz, $CDCl_3$) δ 7.67-7.61 (m, 1H), 7.51-7.39 (m, 5H), 7.35-7.31 (m, 1H), 7.31-7.28 (m, 1H), 7.21-7.15 (m, 1H), 5.51 (s, 2H), 2.72-2.60 (m, 1H), 2.56-2.43 (m, 1H), 2.29-2.17 (m, 1H), 1.96-1.83 (m, 3H).^{13}C NMR (101 MHz, $CDCl_3$) δ 148.56, 142.09, 140.52, 139.94, 136.23, 135.67, 135.62, 130.15, 129.19, 128.97, 128.65, 128.58, 128.42, 128.32 128.29, 127.94, 126.69, 116.11, 98.51, 32.59, 30.79, 26.84.HRMS (ESI) m/z Calcd. for $C_{22}H_{18}N_3O_2^+$([M+H]$^+$)356.1394,Found 356.1400.

4. -isocyano-2-nitro-1-phenyl-9H-fluoren-3-amine (7ai)

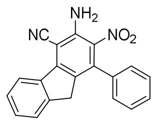

7ai

Prepared according to the general procedure as yellow solid (8 mg, 13% yield) after silica gel chromatography (petroleum ether/EtOAc = 10:1). ^{1}H NMR (400 MHz, DMSO) δ 8.27 (dd, *J* = 7.1, 1.7 Hz, 1H), 7.55 (d, *J* = 7.5 Hz, 1H), 7.50-7.39 (m, 5H), 7.36-7.30 (m, 2H), 6.63 (s, 2H), 2.45 (p, *J* = 1.8 Hz, 12H). ^{13}C NMR (101 MHz, DMSO) δ 146.76, 145.52, 144.54, 138.00, 137.77, 135.56, 135.51, 131.24, 130.09, 129.26, 129.11, 128.05, 127.92, 126.06, 122.33, 116.17, 89.58, 36.12.HRMS (ESI) m/z Calcd. for $C_{20}H_{14}N_3O_2^+$([M+H]$^+$)328.1081,Found 328.1090.

3. NMR spectra for compounds

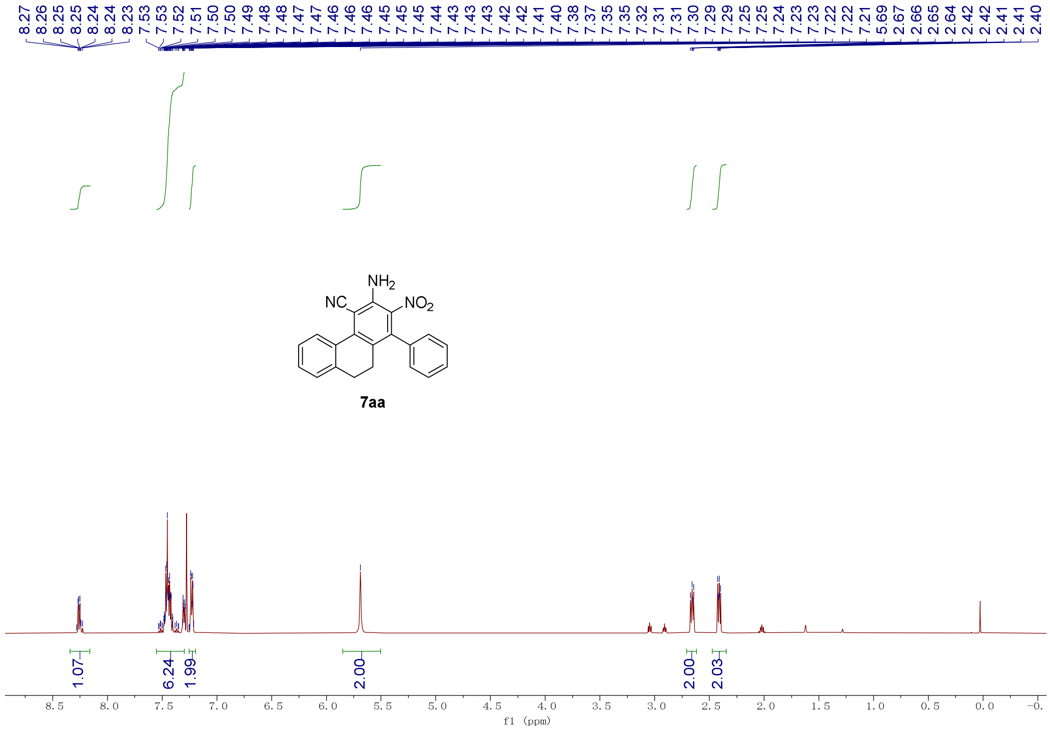

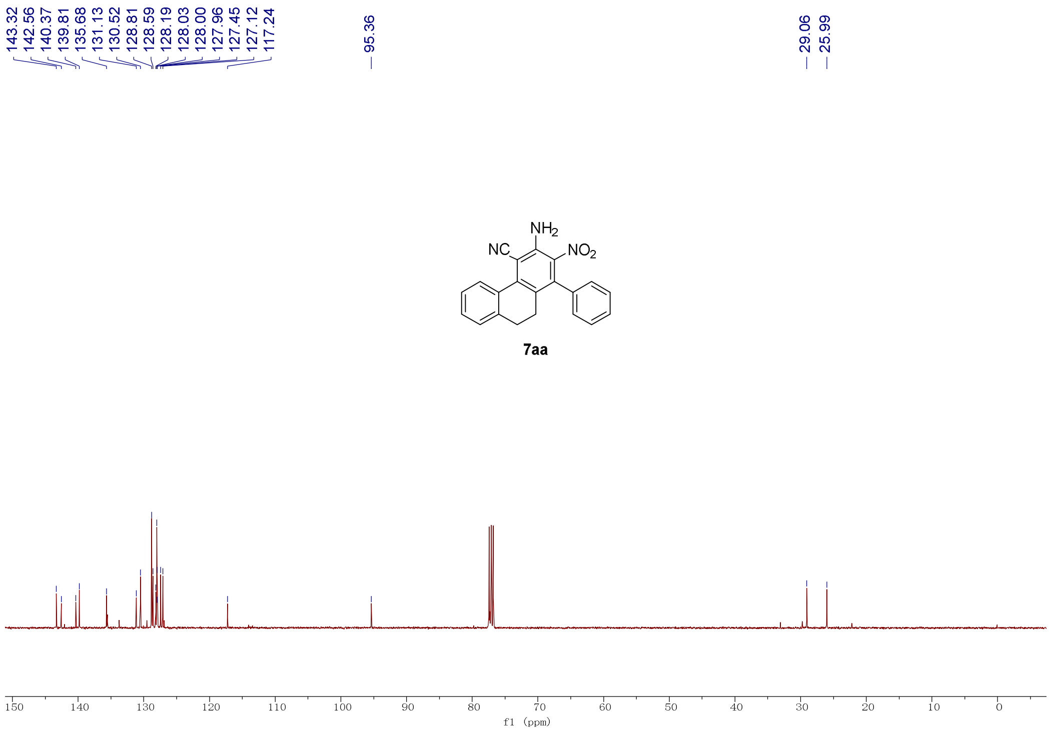
143.32
142.56
140.37
139.81
135.68
131.13
130.52
128.81
128.59
128.19
128.03
128.00
127.96
127.45
127.12
117.24
95.36
29.06
25.99
NH2
NC
NO2
7aa
f1 (ppm)

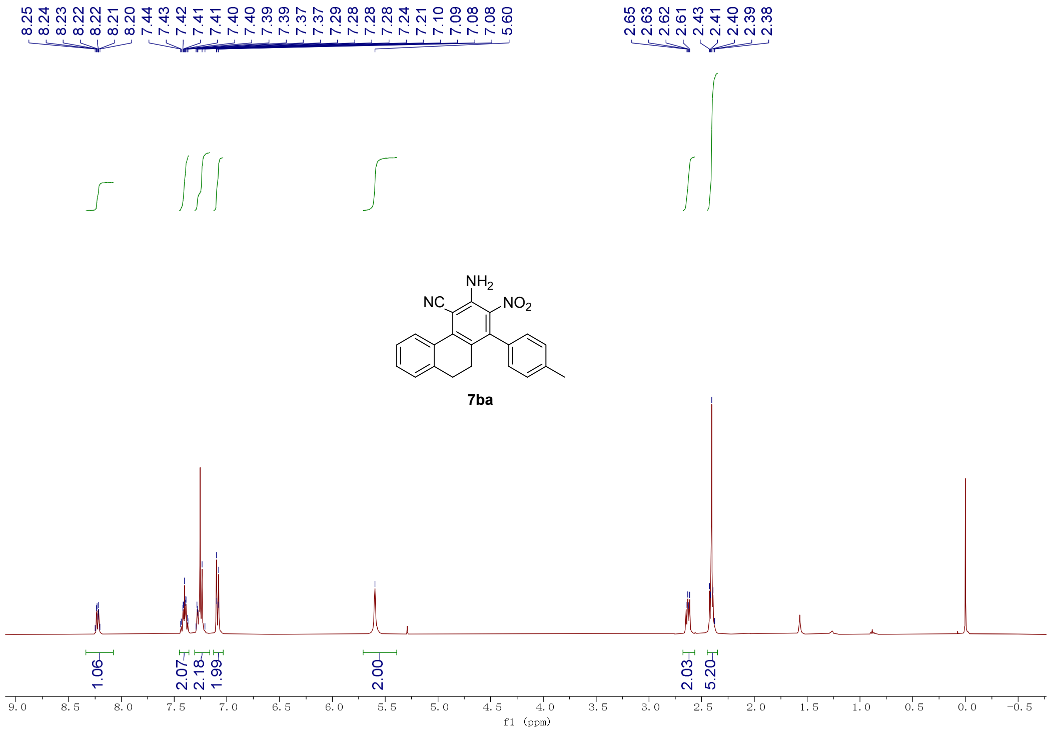
8.25
8.24
8.23
8.22
8.22
8.21
8.20
7.44
7.43
7.42
7.41
7.41
7.40
7.40
7.39
7.39
7.37
7.37
7.29
7.28
7.28
7.28
7.24
7.21
7.10
7.09
7.08
7.08
5.60
2.65
2.63
2.62
2.61
2.43
2.41
2.40
2.39
2.38
NH2
NC
NO2
7ba
1.06
2.07
2.18
1.99
2.00
2.03
5.20
f1 (ppm)

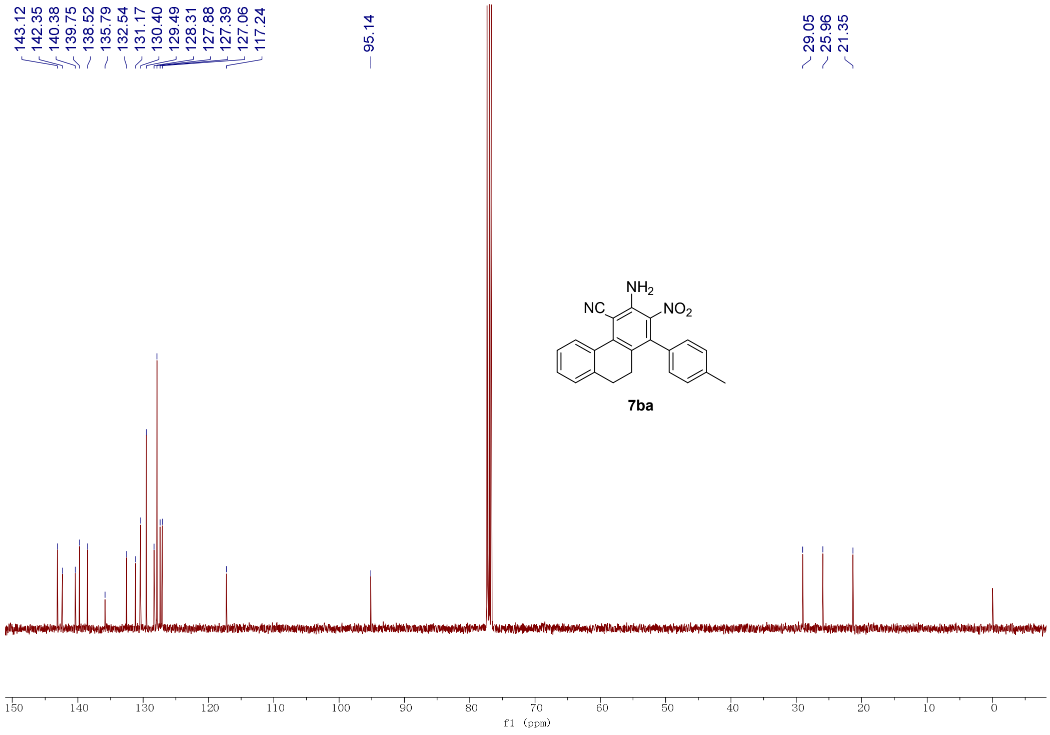
143.12
142.35
140.38
139.75
138.52
135.79
132.54
131.17
130.40
129.49
128.31
127.88
127.39
127.06
117.24
95.14
29.05
25.96
21.35
NH2
NC
NO2
7ba
f1 (ppm)

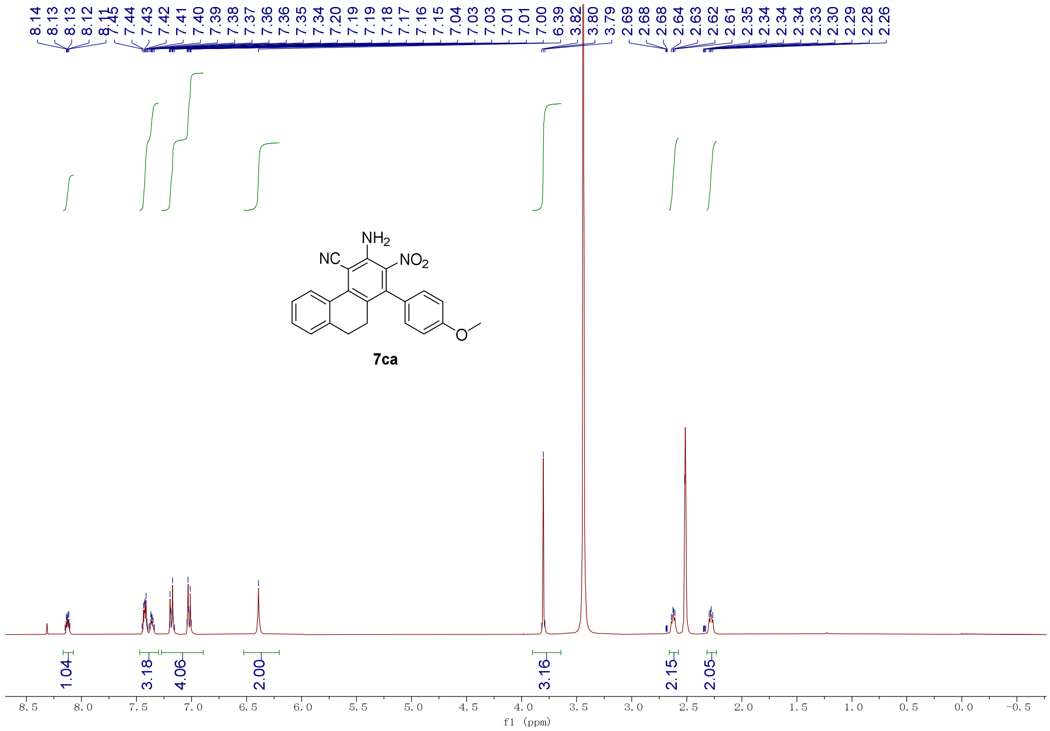
8.14 8.13 8.13 8.12 8.11 7.45 7.44 7.43 7.42 7.41 7.40 7.39 7.38 7.37 7.36 7.36 7.35 7.34 7.20 7.19 7.19 7.18 7.17 7.16 7.15 7.04 7.03 7.03 7.01 7.01 7.00 6.39 3.82 3.80 3.79 2.69 2.68 2.68 2.64 2.63 2.62 2.61 2.35 2.34 2.34 2.34 2.33 2.30 2.29 2.28 2.26
NH2
NC
NO2
O
7ca
1.04 3.18 4.06 2.00 3.16 2.15 2.05
8.5 8.0 7.5 7.0 6.5 6.0 5.5 5.0 4.5 4.0 3.5 3.0 2.5 2.0 1.5 1.0 0.5 0.0 -0.5
f1 (ppm)

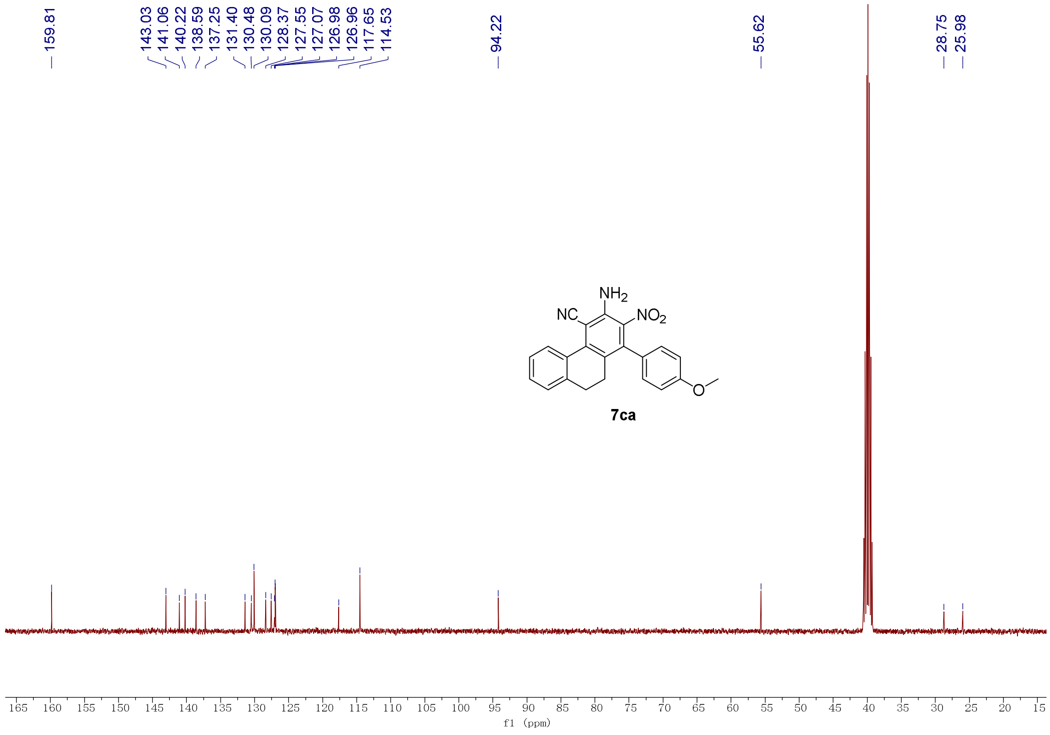
159.81 143.03 141.06 140.22 138.59 137.25 131.40 130.48 130.09 128.37 127.55 127.07 126.98 126.96 117.65 114.53 94.22 55.62 28.75 25.98
NH2
NC
NO2
O
7ca
165 160 155 150 145 140 135 130 125 120 115 110 105 100 95 90 85 80 75 70 65 60 55 50 45 40 35 30 25 20 15
f1 (ppm)

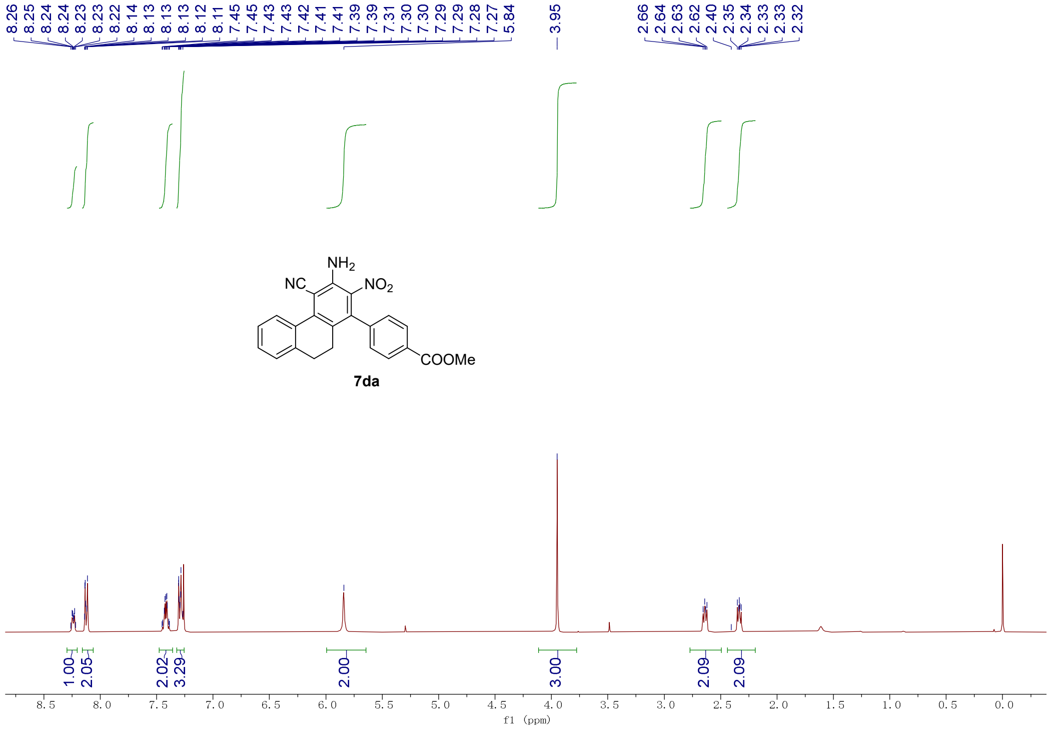
8.26
8.25
8.24
8.24
8.23
8.23
8.22
8.14
8.13
8.13
8.13
8.12
8.11
7.45
7.45
7.43
7.43
7.42
7.41
7.41
7.39
7.31
7.30
7.29
7.29
7.28
7.27
5.84
3.95
2.66
2.64
2.63
2.62
2.40
2.35
2.34
2.33
2.33
2.32
NH2
NC
NO2
COOMe
7da
1.00
2.05
2.02
3.29
2.00
3.00
2.09
2.09
8.5
8.0
7.5
7.0
6.5
6.0
5.5
5.0
4.5
4.0
3.5
3.0
2.5
2.0
1.5
1.0
0.5
0.0
f1 (ppm)

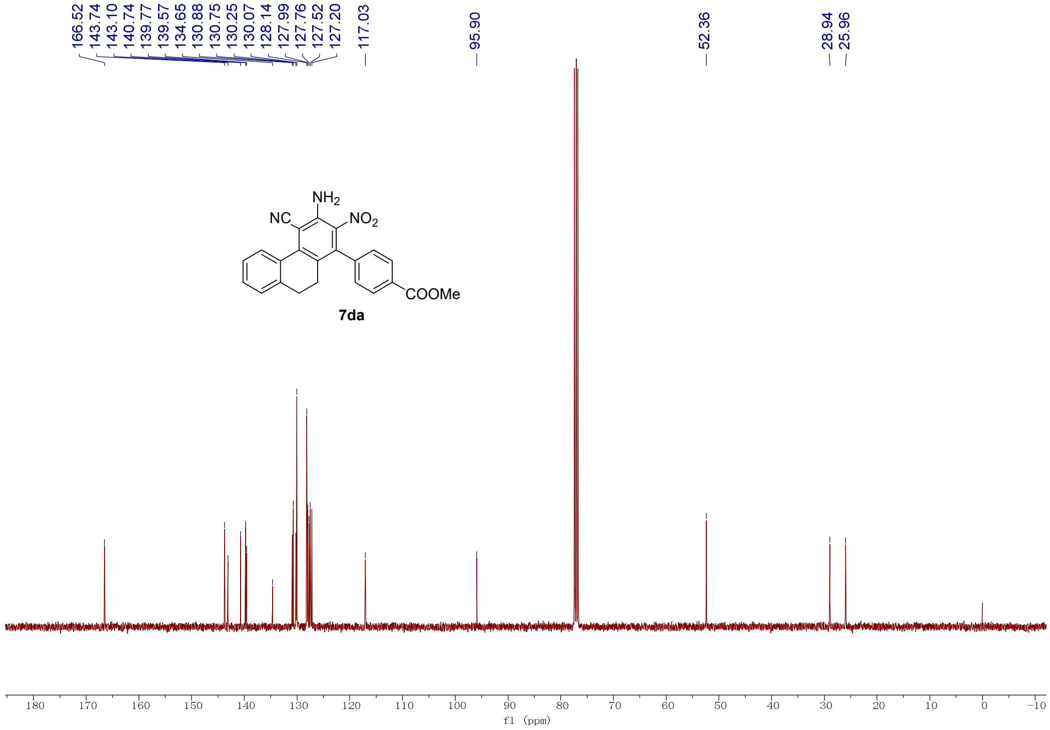
166.52
143.74
143.10
140.74
139.77
139.57
134.65
130.88
130.75
130.25
130.07
128.14
127.99
127.76
127.52
127.20
117.03
95.90
52.36
28.94
25.96
NH2
NC
NO2
COOMe
7da
180
170
160
150
140
130
120
110
100
90
80
70
60
50
40
30
20
10
0
−10
f1 (ppm)

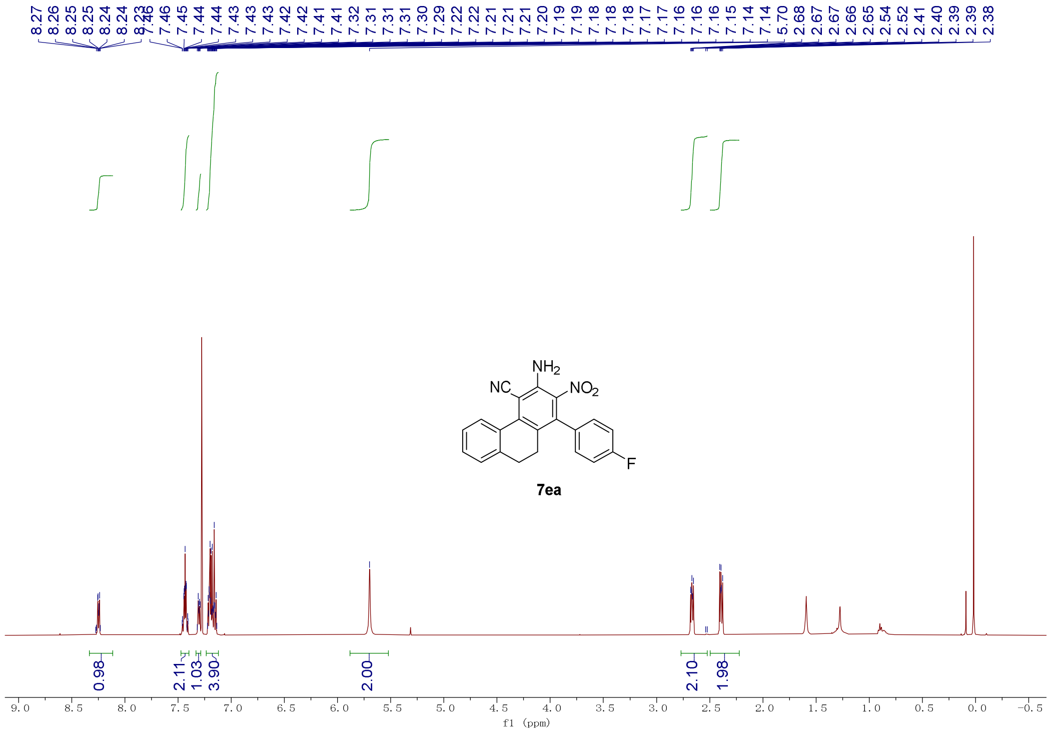

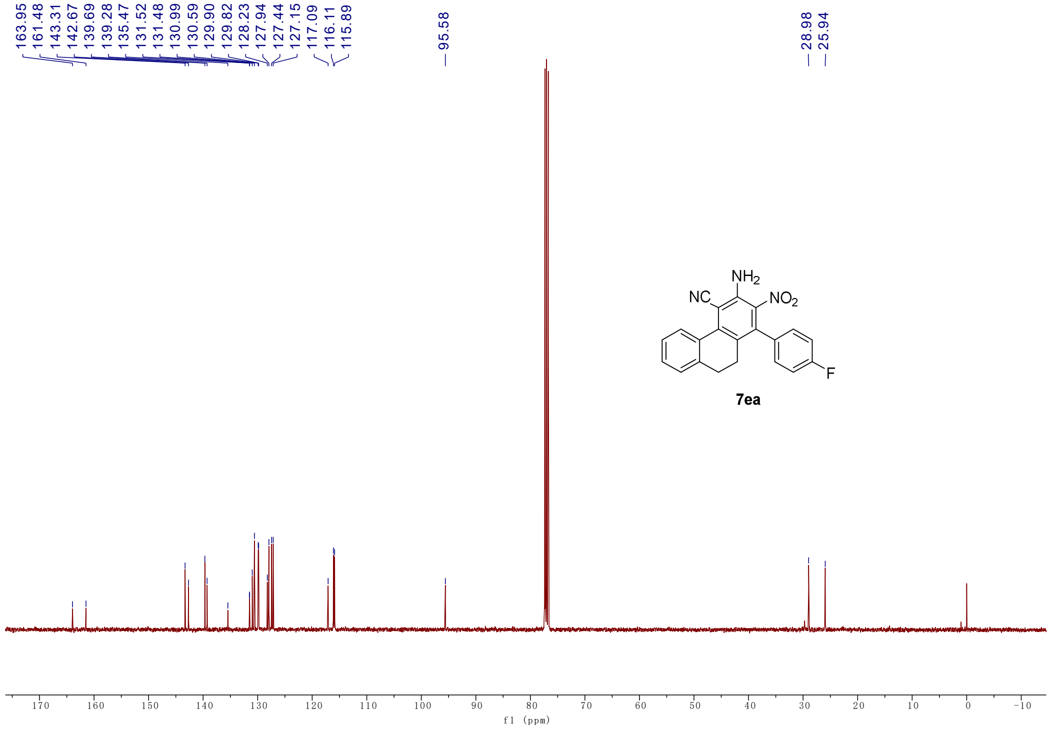

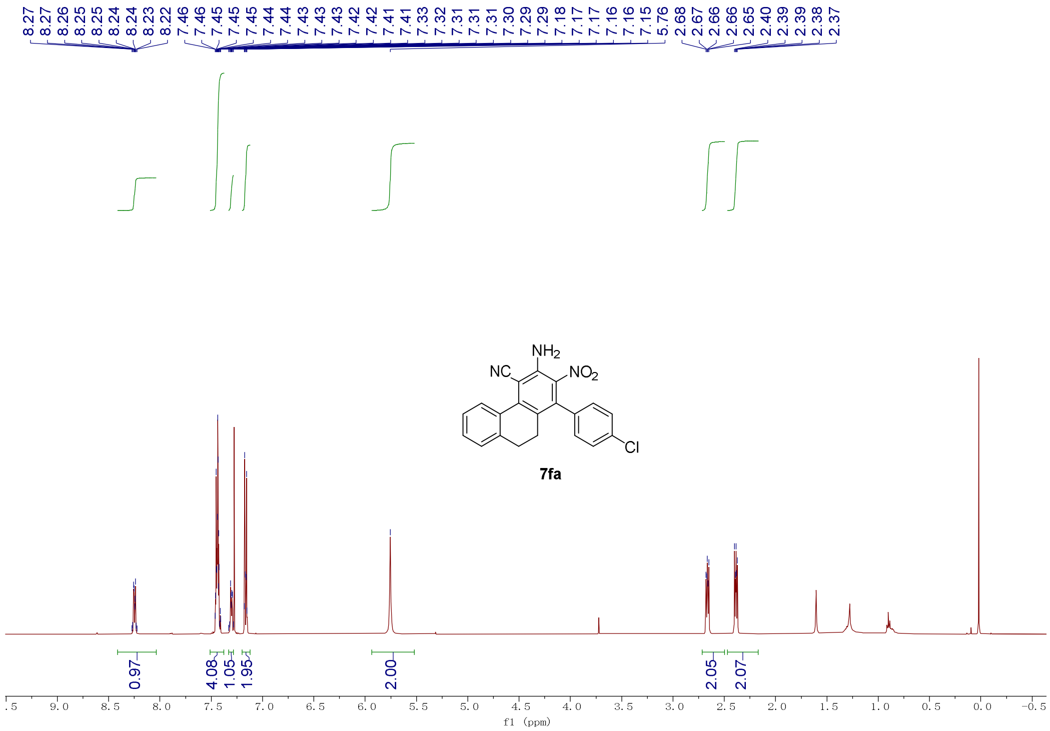

7fa

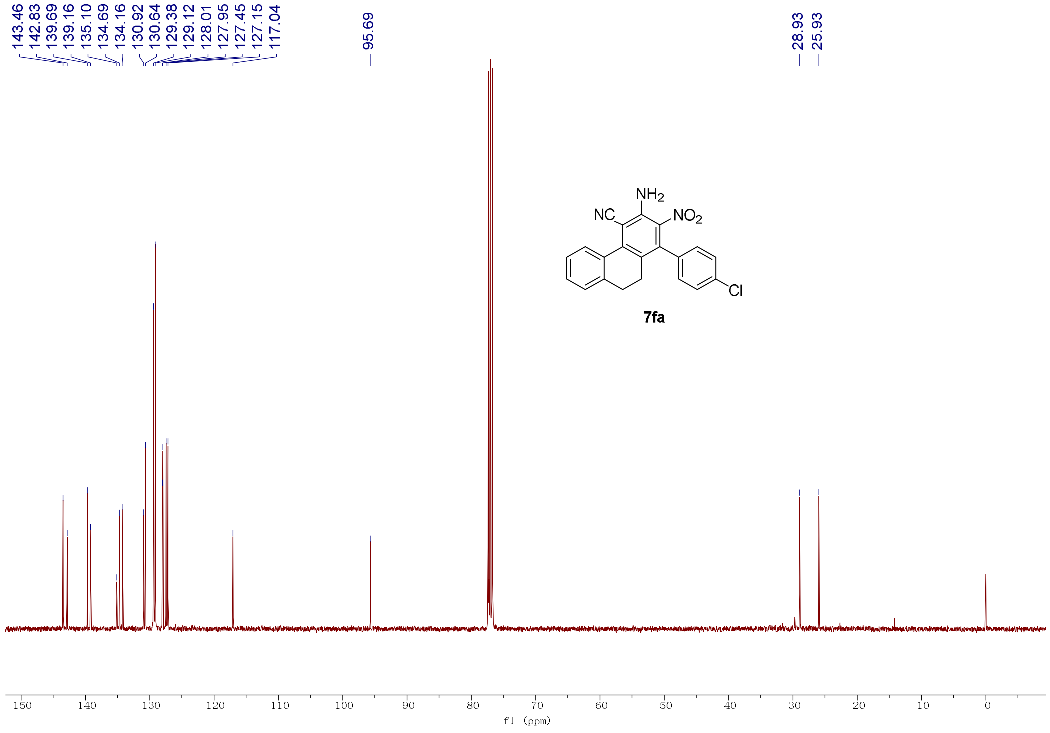

7fa

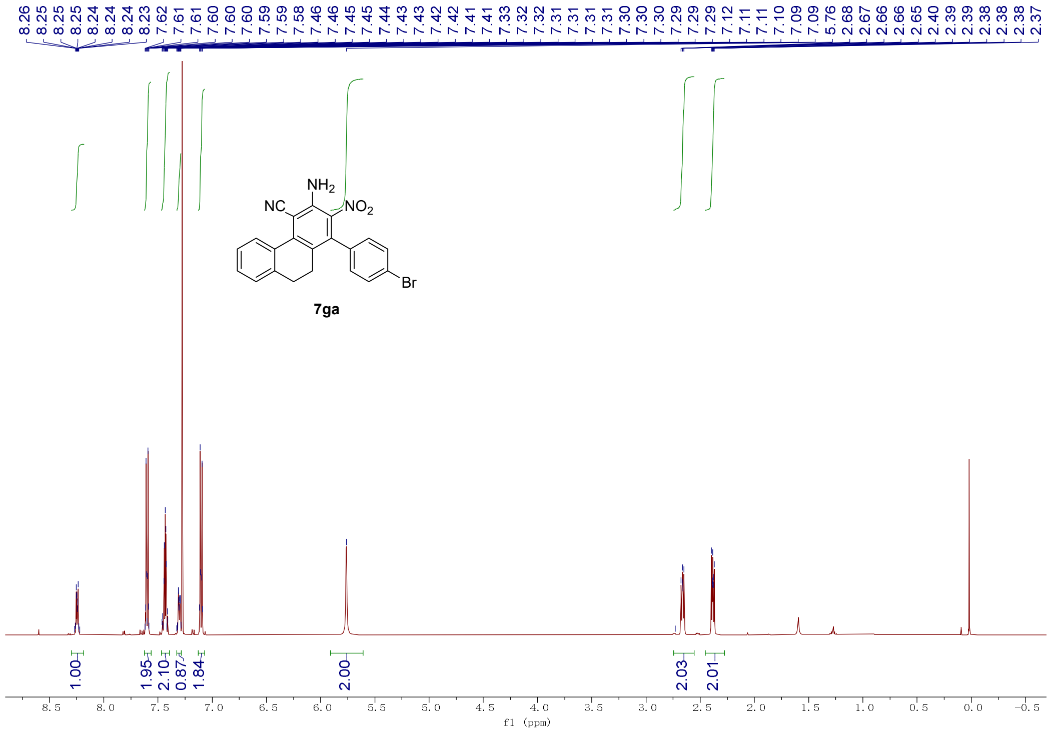
8.26
8.25
8.25
8.24
8.24
8.24
8.23
7.62
7.61
7.61
7.60
7.60
7.59
7.59
7.58
7.46
7.46
7.45
7.45
7.44
7.44
7.43
7.43
7.42
7.42
7.41
7.41
7.33
7.32
7.32
7.31
7.31
7.31
7.31
7.30
7.30
7.30
7.29
7.29
7.29
7.12
7.11
7.11
7.10
7.09
7.09
5.76
2.68
2.67
2.66
2.65
2.40
2.39
2.39
2.38
2.38
2.38
2.38
2.37
NH2
NC
NO2
Br
7ga
1.00
1.95
2.10
0.87
1.84
2.00
2.03
2.01
f1 (ppm)

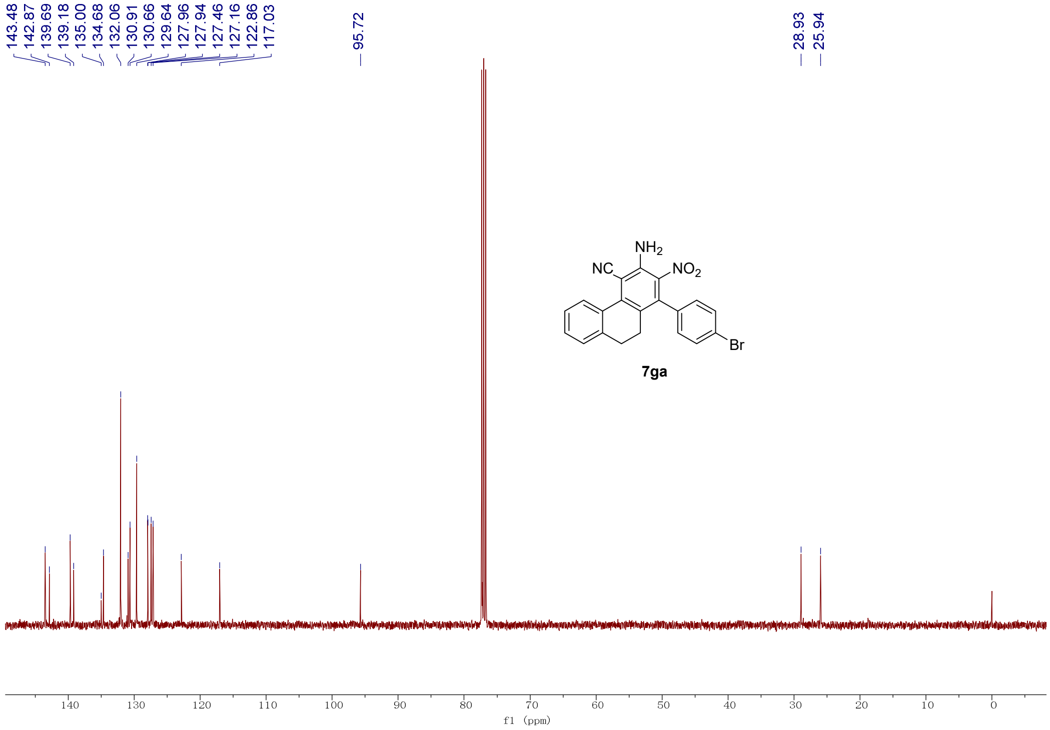
143.48
142.87
139.69
139.18
135.00
134.68
132.06
130.91
130.66
129.64
127.96
127.94
127.46
127.16
122.86
117.03
95.72
28.93
25.94
NH2
NC
NO2
Br
7ga
f1 (ppm)

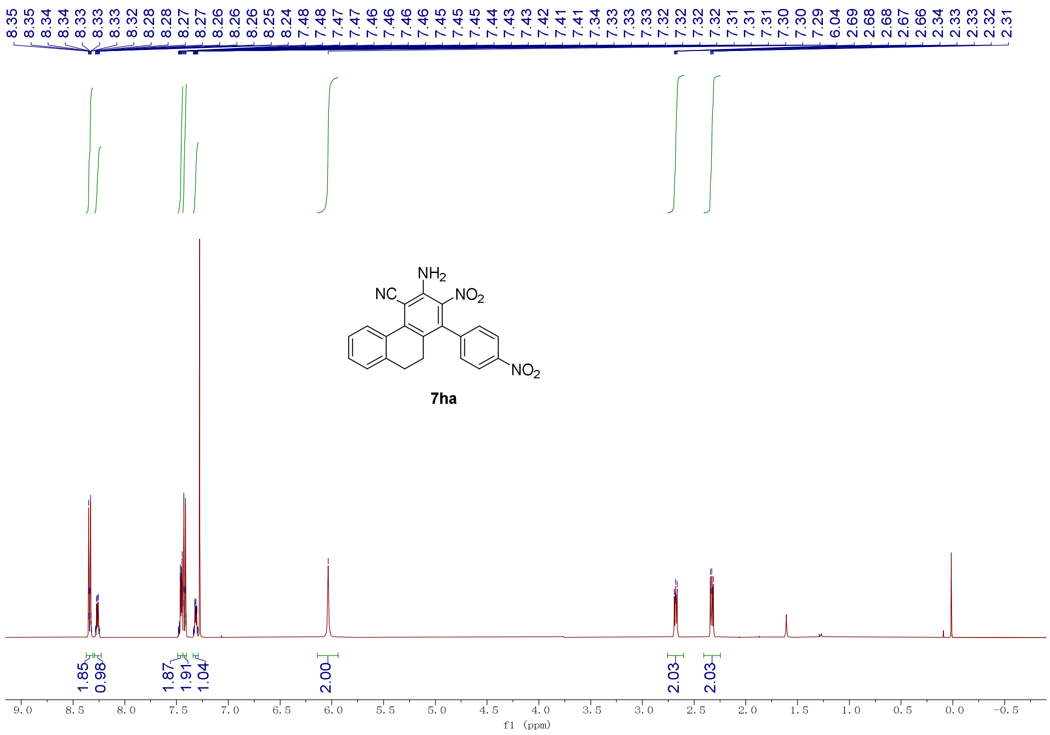

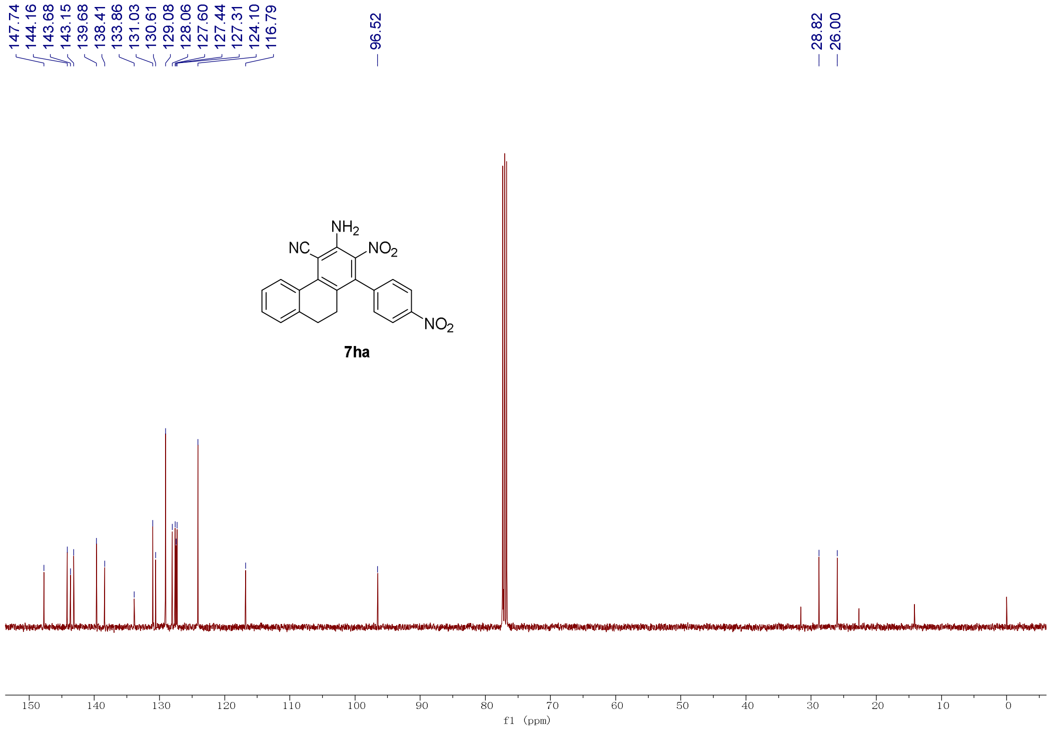

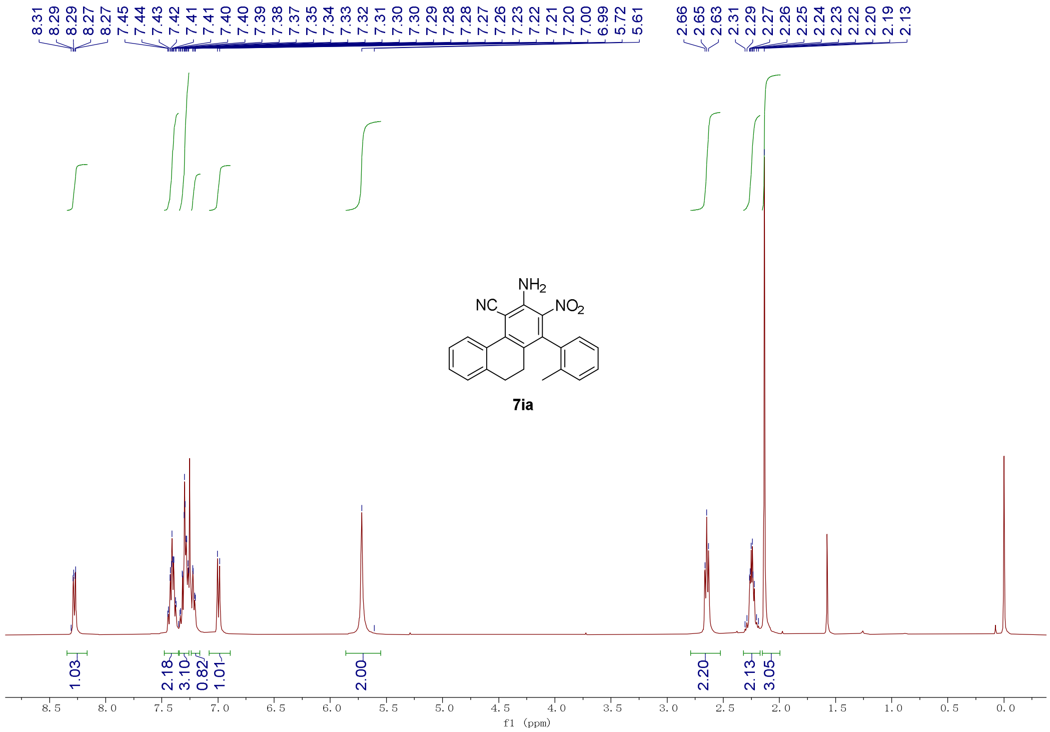
8.31
8.29
8.29
8.27
7.45
7.44
7.43
7.42
7.41
7.41
7.40
7.40
7.39
7.38
7.37
7.35
7.34
7.33
7.32
7.31
7.30
7.30
7.29
7.28
7.28
7.27
7.26
7.23
7.22
7.21
7.20
7.00
6.99
5.72
5.61
2.66
2.65
2.63
2.31
2.29
2.27
2.26
2.25
2.24
2.23
2.22
2.20
2.19
2.13
NH2
NC
NO2
7ia
1.03
2.18
3.10
0.82
1.01
2.00
2.20
2.13
3.05
f1 (ppm)

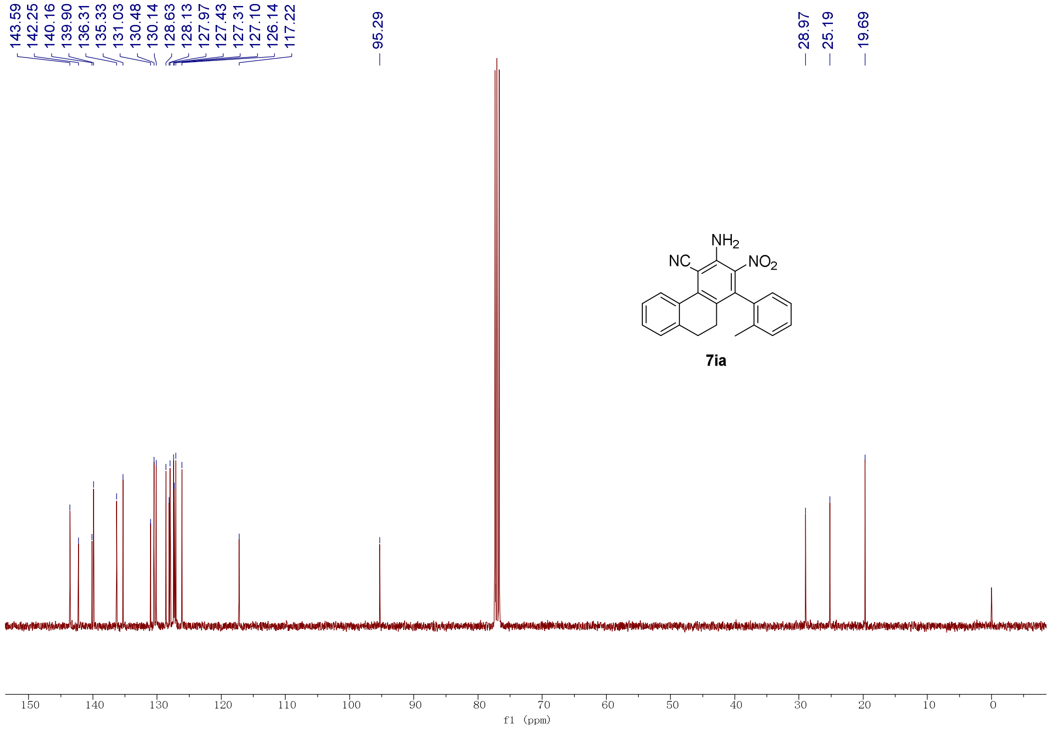
143.59
142.25
140.16
139.90
136.31
135.33
131.03
130.48
130.14
128.63
128.13
127.97
127.43
127.31
127.10
126.14
117.22
95.29
28.97
25.19
19.69
NH2
NC
NO2
7ia
f1 (ppm)

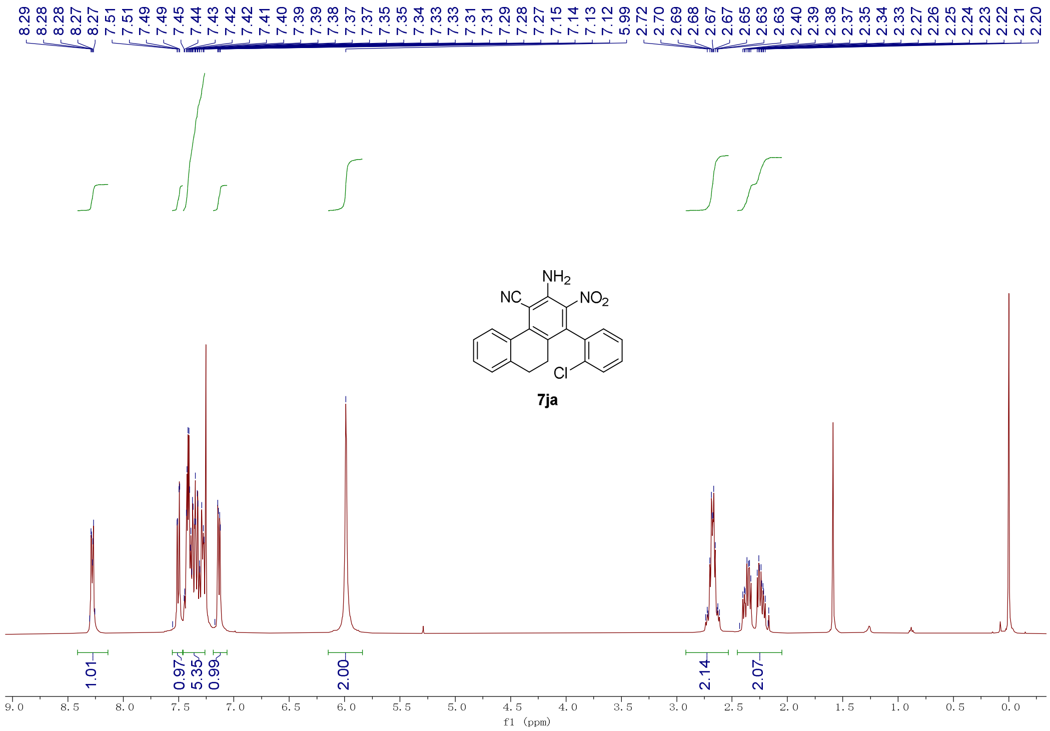

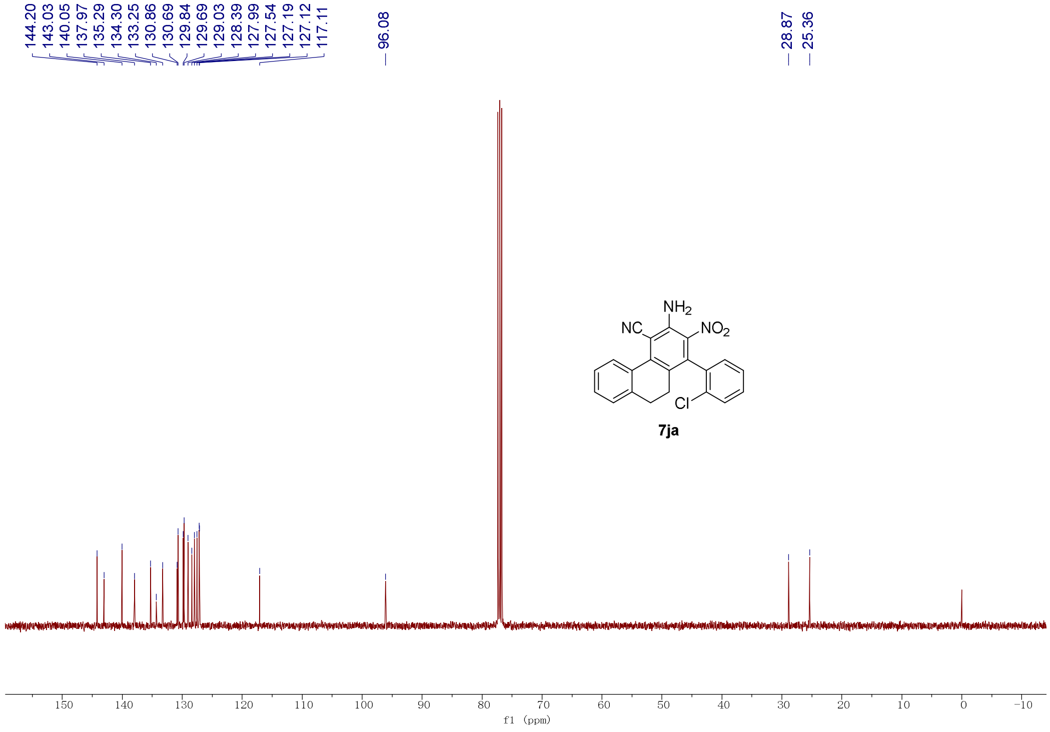

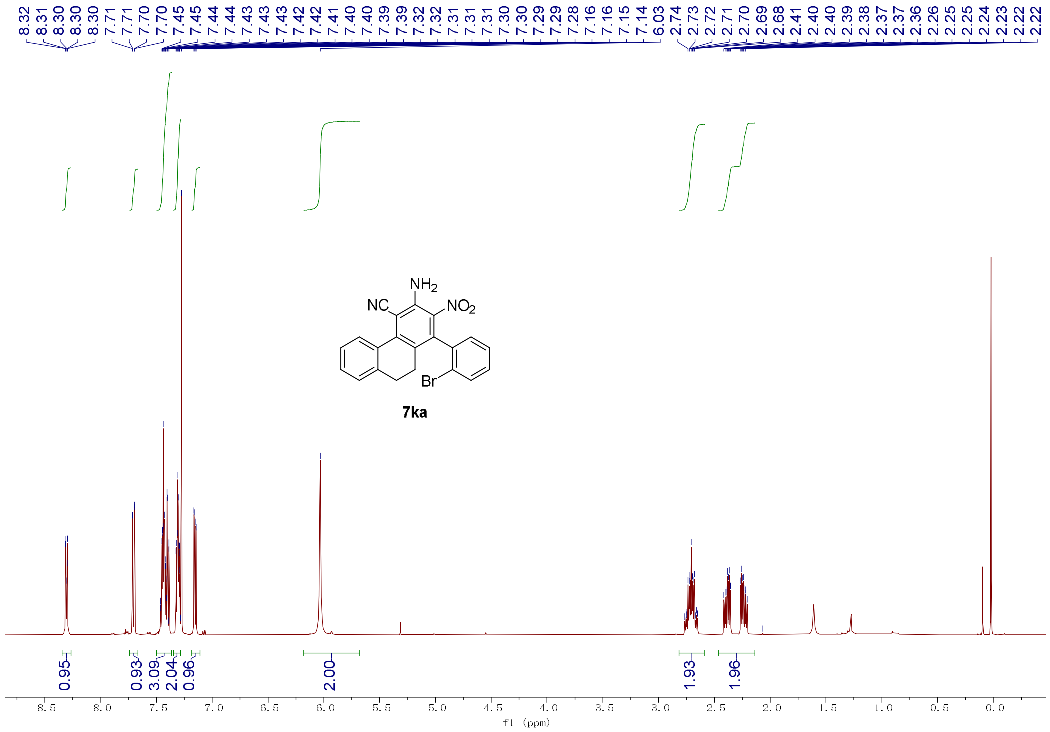
8.32 8.31 8.30 8.30 8.30 7.71 7.71 7.70 7.70 7.45 7.45 7.44 7.44 7.43 7.43 7.43 7.42 7.42 7.41 7.40 7.40 7.39 7.39 7.32 7.32 7.31 7.31 7.31 7.30 7.30 7.29 7.29 7.28 7.16 7.16 7.15 7.14 6.03 2.74 2.73 2.72 2.71 2.70 2.69 2.68 2.41 2.40 2.40 2.39 2.38 2.37 2.37 2.36 2.26 2.25 2.25 2.24 2.23 2.22 2.22
NH2
NC
NO2
Br
7ka
0.95 0.93 3.09 2.04 0.96 2.00 1.93 1.96
f1 (ppm)

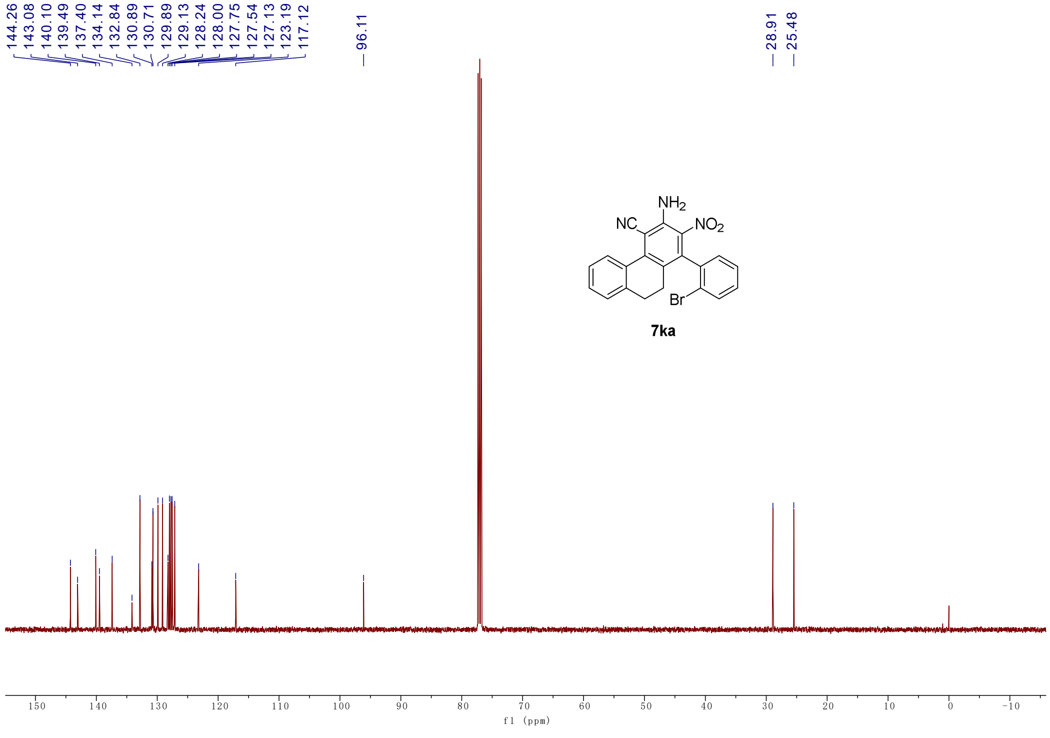
144.26 143.08 140.10 139.49 137.40 134.14 132.84 130.89 130.71 129.89 129.13 128.24 128.00 127.75 127.54 127.13 123.19 117.12
96.11
28.91 25.48
NH2
NC
NO2
Br
7ka
f1 (ppm)

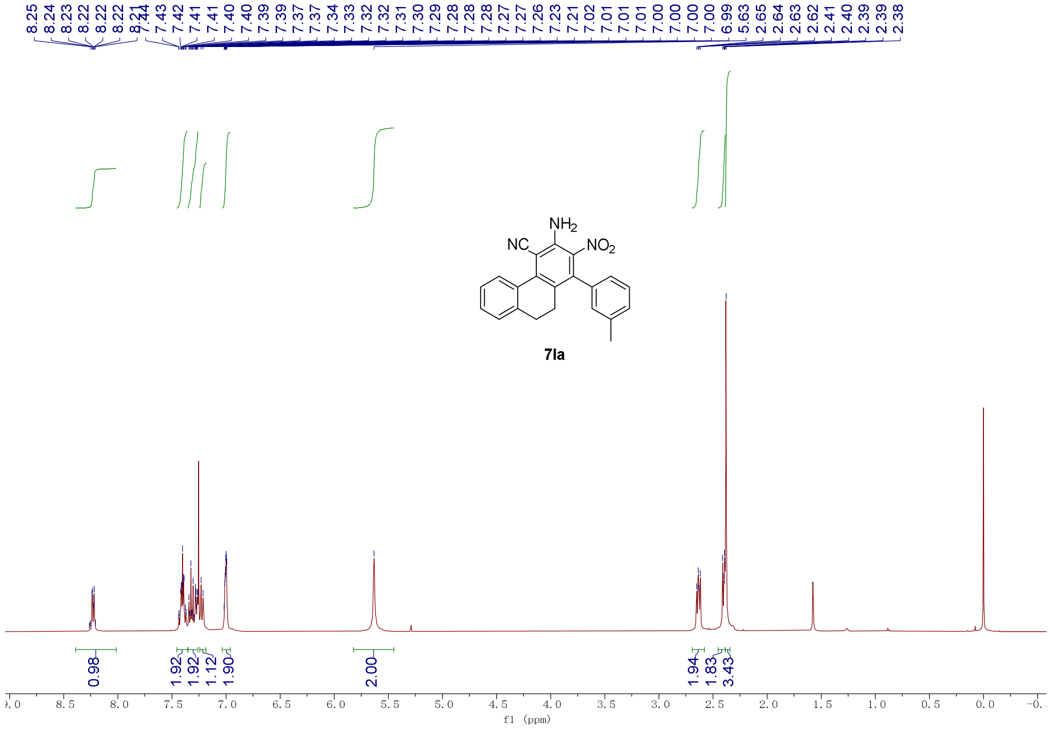
NH2
NC
NO2
7la
f1 (ppm)

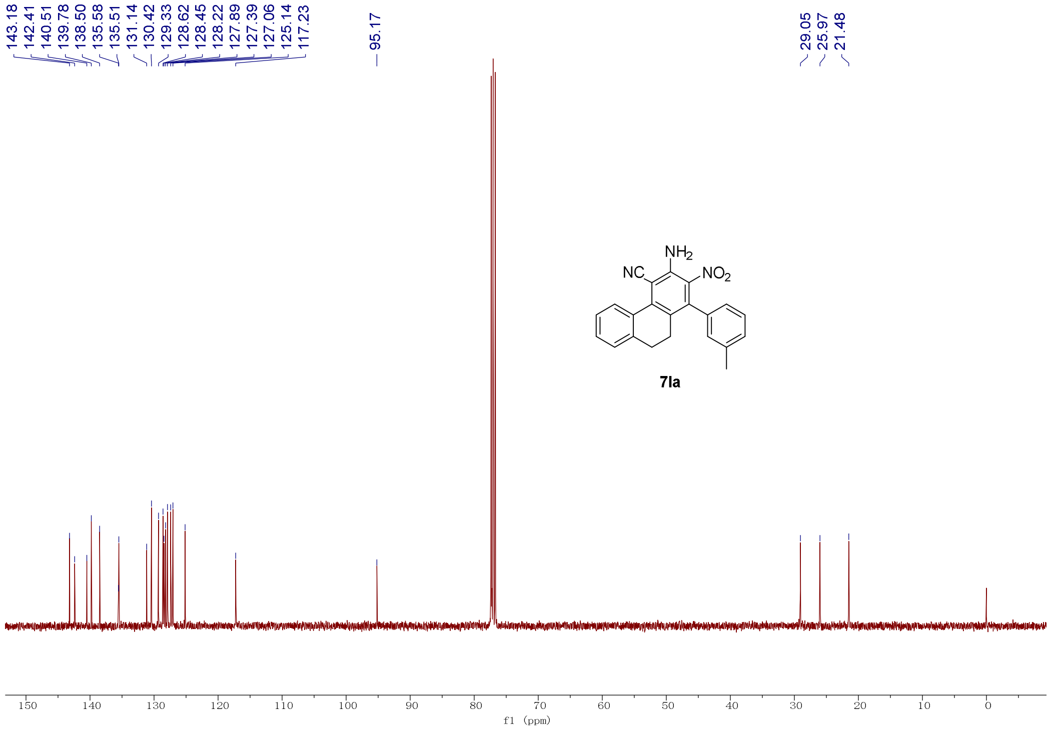
NH2
NC
NO2
7la
f1 (ppm)

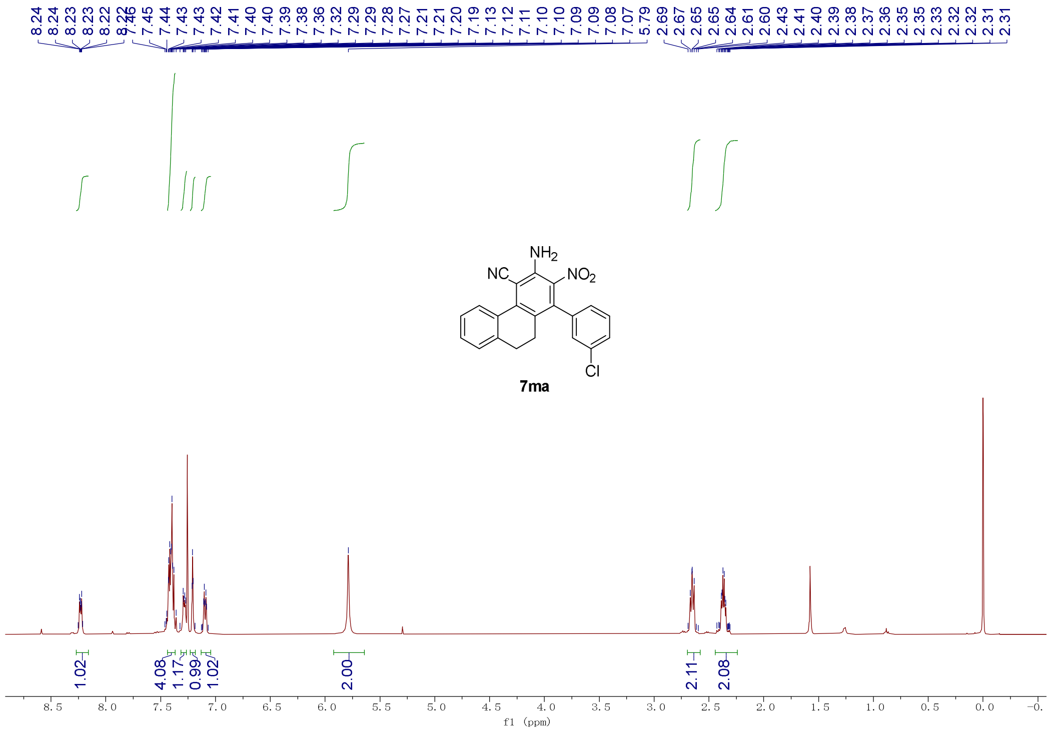

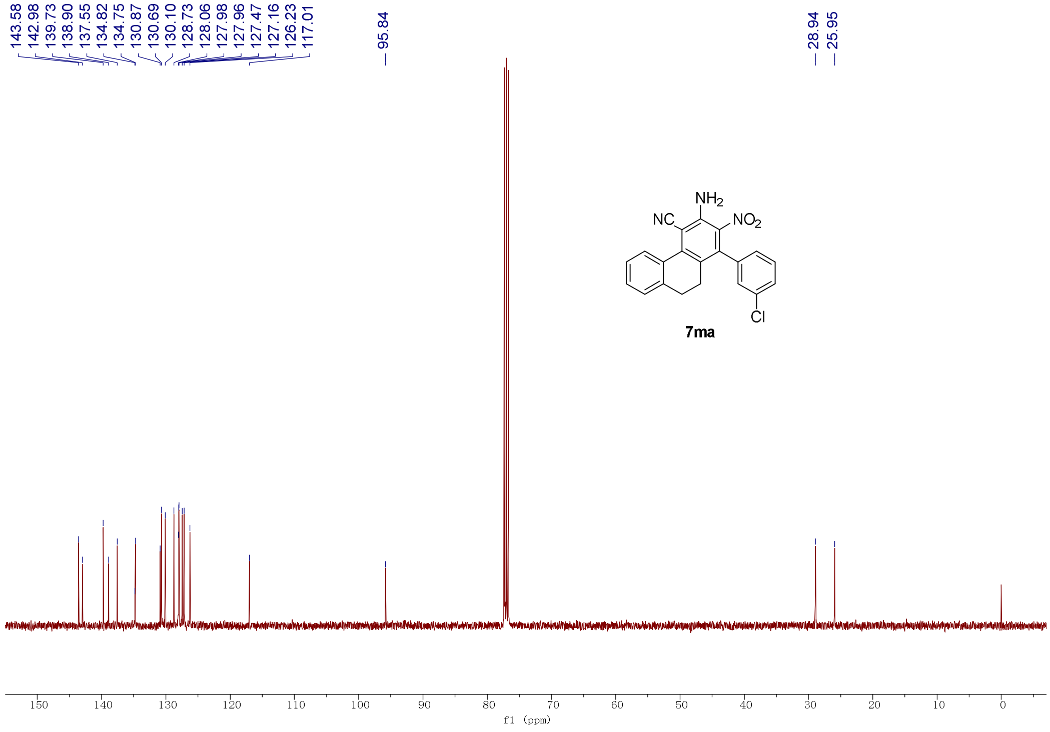

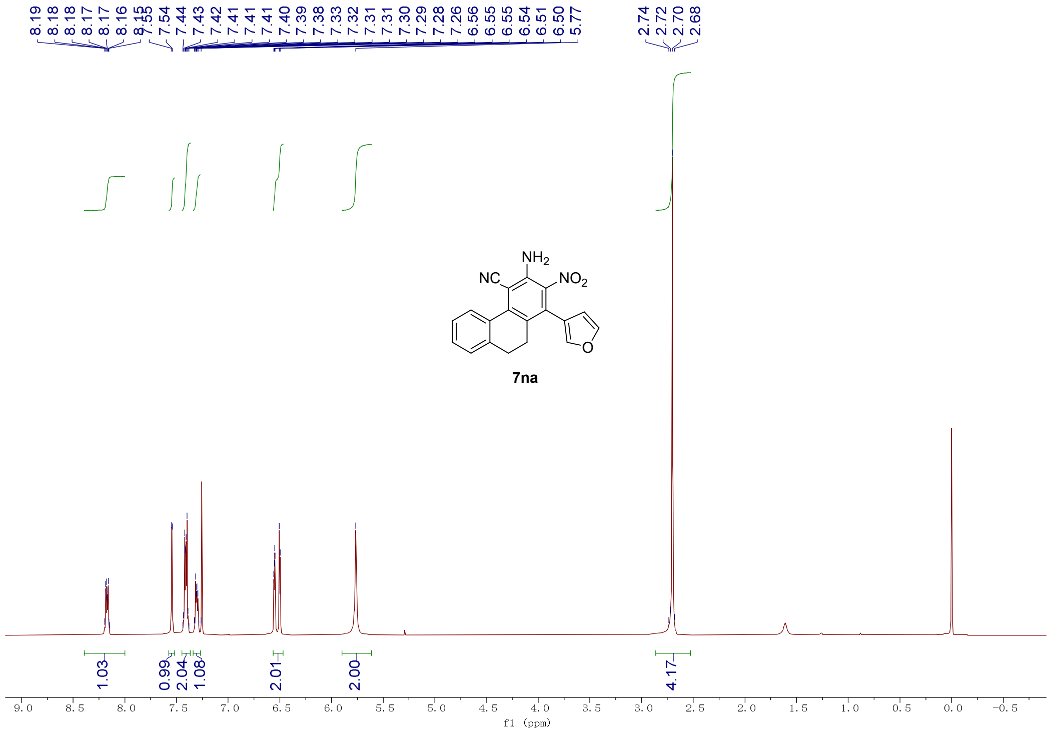
8.19
8.18
8.18
8.17
8.17
8.16
8.15
7.55
7.54
7.44
7.43
7.42
7.41
7.41
7.41
7.40
7.39
7.38
7.33
7.32
7.31
7.31
7.30
7.29
7.28
7.26
6.56
6.55
6.55
6.54
6.51
6.50
5.77
2.74
2.72
2.70
2.68
NH2
NC
NO2
O
7na
1.03
0.99
2.04
1.08
2.01
2.00
4.17
9.0 8.5 8.0 7.5 7.0 6.5 6.0 5.5 5.0 4.5 4.0 3.5 3.0 2.5 2.0 1.5 1.0 0.5 0.0 -0.5
f1 (ppm)

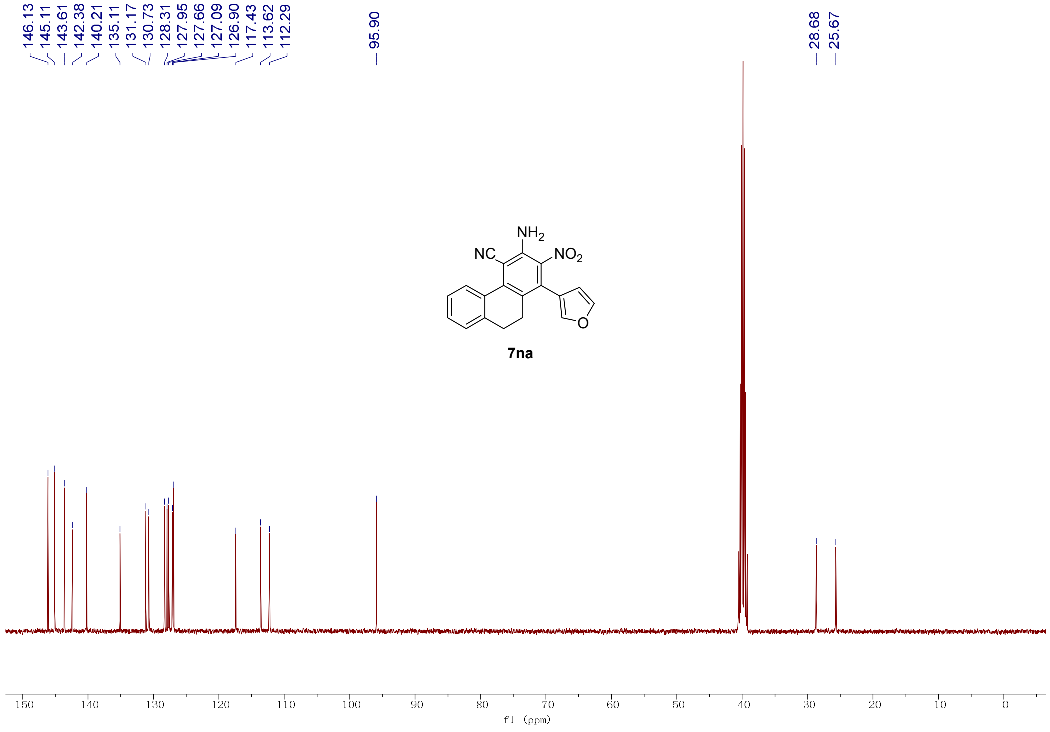
146.13
145.11
143.61
142.38
140.21
135.11
131.17
130.73
128.31
127.95
127.66
127.09
126.90
117.43
113.62
112.29
95.90
28.68
25.67
NH2
NC
NO2
O
7na
150 140 130 120 110 100 90 80 70 60 50 40 30 20 10 0
f1 (ppm)

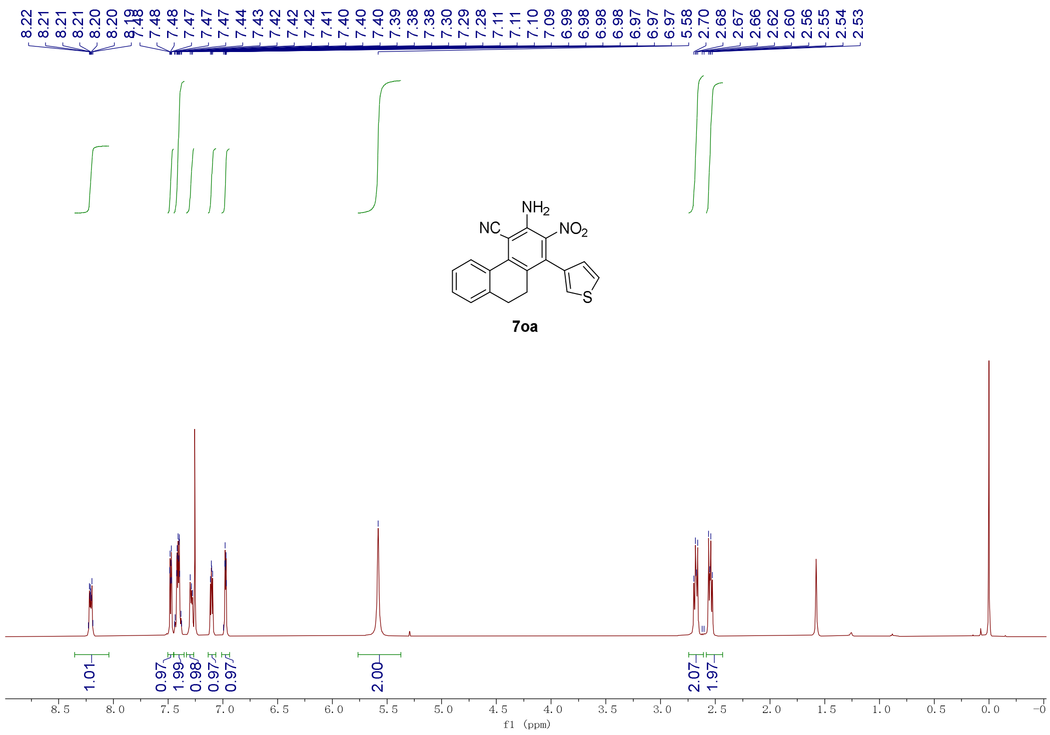
8.22 8.21 8.21 8.20 8.20 8.19 7.48 7.48 7.47 7.47 7.47 7.44 7.43 7.42 7.42 7.41 7.40 7.40 7.40 7.39 7.38 7.38 7.30 7.29 7.28 7.11 7.11 7.10 7.09 6.99 6.98 6.98 6.98 6.97 6.97 5.58 2.70 2.68 2.67 2.66 2.62 2.60 2.56 2.55 2.54 2.53
NH2
NC
NO2
S
7oa
1.01 0.97 1.99 0.98 0.97 0.97 2.00 2.07 1.97
f1 (ppm)

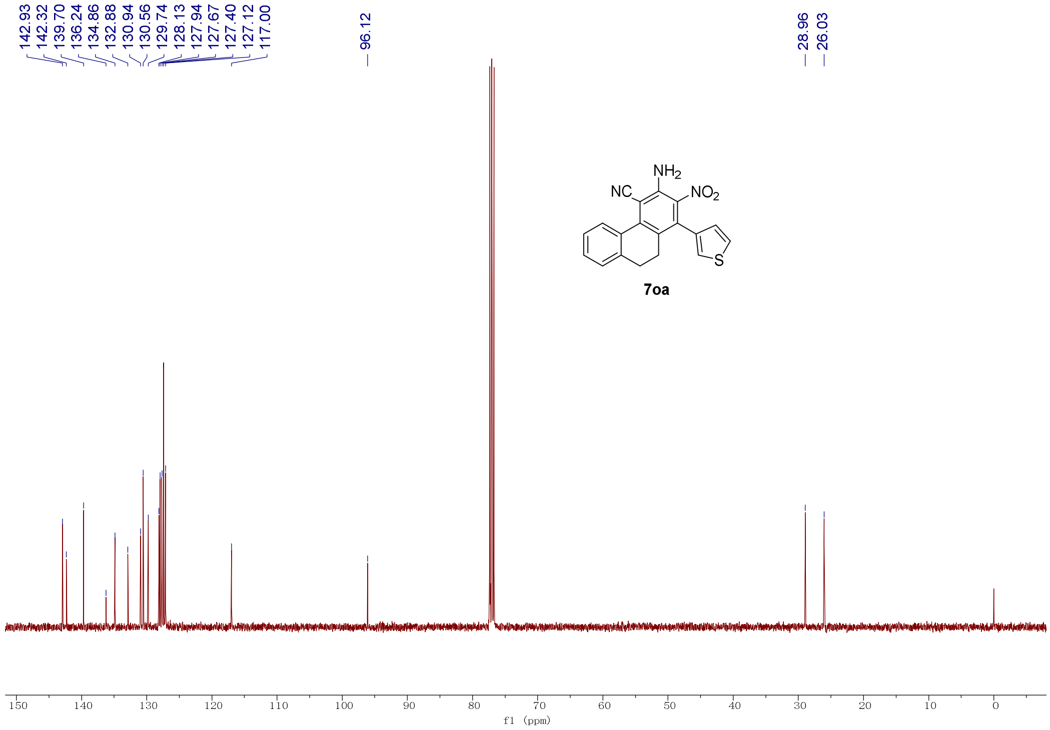
142.93 142.32 139.70 136.24 134.86 132.88 130.94 130.56 129.74 128.13 127.94 127.67 127.40 127.12 117.00
96.12
28.96 26.03
NH2
NC
NO2
S
7oa
f1 (ppm)

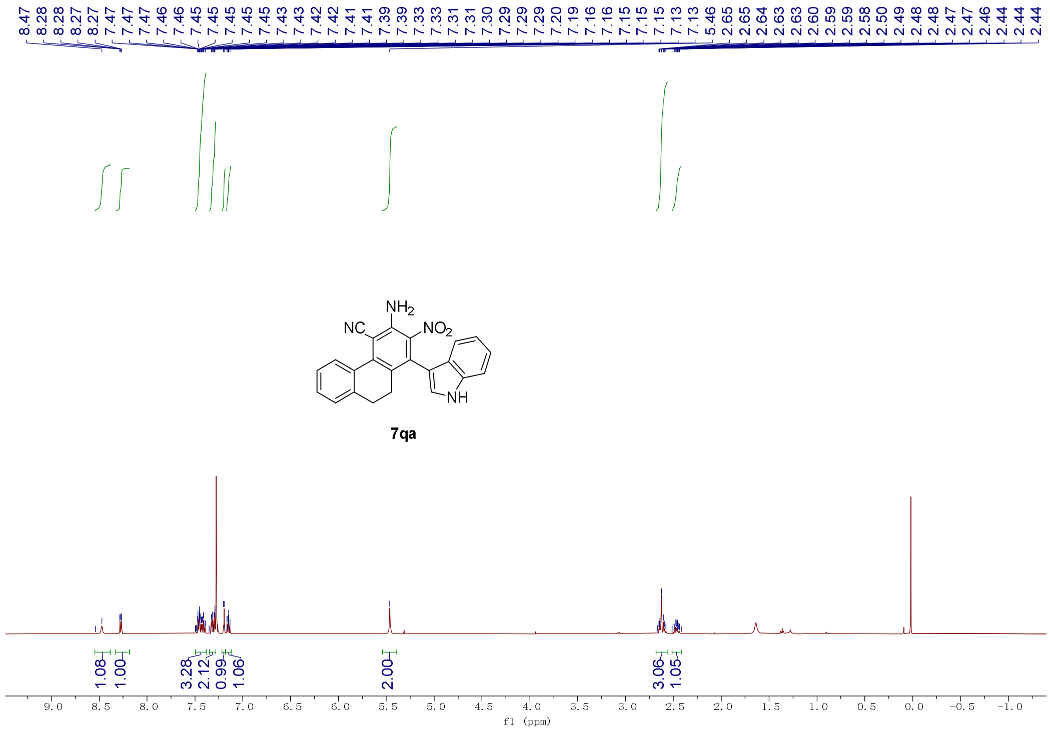

7qa

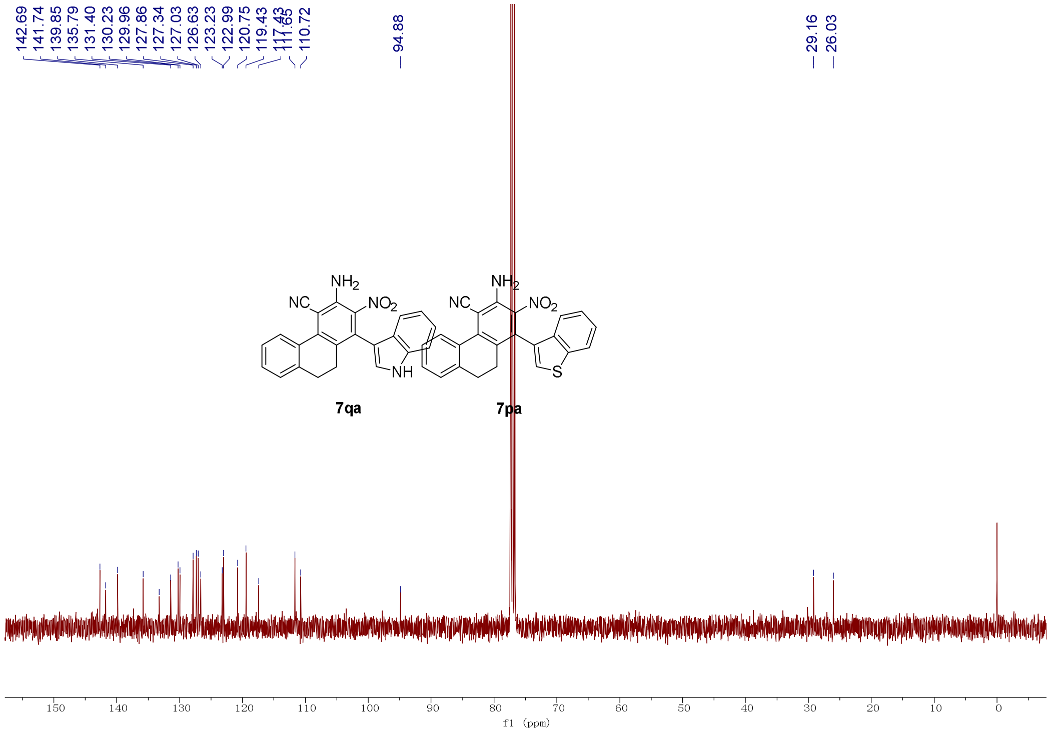
142.69
141.74
139.85
135.79
131.40
130.23
129.96
127.86
127.34
127.03
126.63
123.23
122.99
120.75
119.43
117.43
111.65
110.72
94.88
29.16
26.03
NH2
NC
NO2
NH
S
7qa
7pa
f1 (ppm)

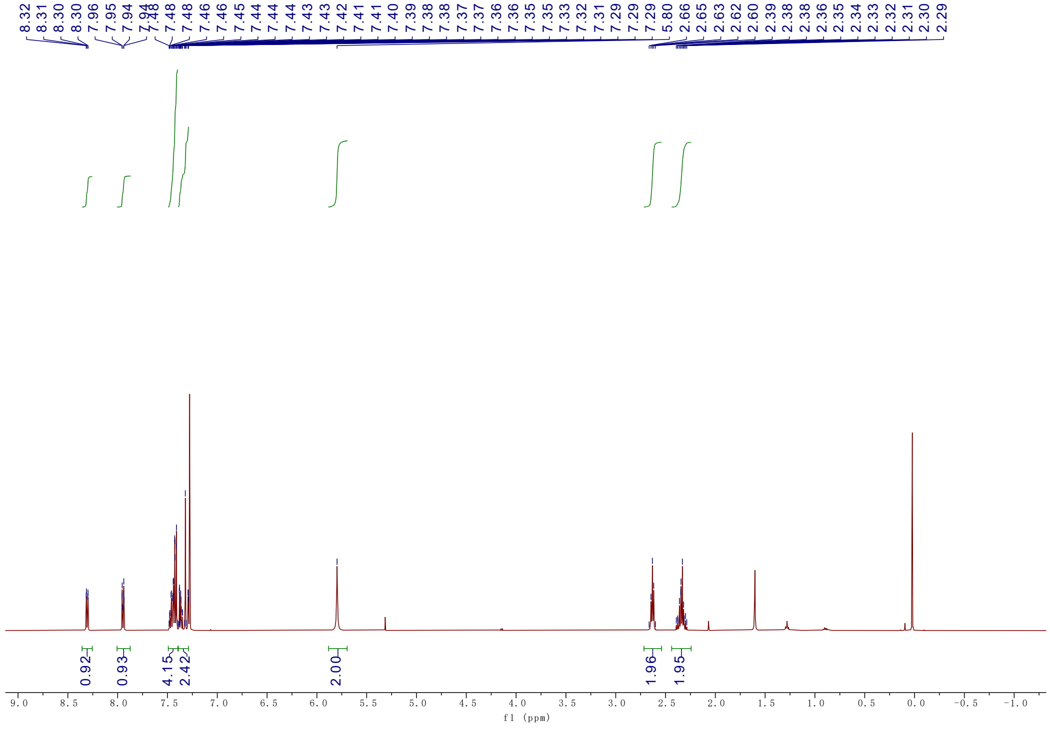
8.32
8.31
8.30
8.30
7.96
7.95
7.94
7.94
7.48
7.48
7.46
7.45
7.44
7.44
7.43
7.42
7.41
7.40
7.39
7.38
7.38
7.37
7.37
7.36
7.36
7.35
7.35
7.33
7.32
7.31
7.29
7.29
7.29
5.80
2.66
2.65
2.63
2.62
2.60
2.39
2.38
2.38
2.36
2.35
2.34
2.33
2.32
2.31
2.30
2.29
0.92
0.93
4.15
2.42
2.00
1.96
1.95
f1 (ppm)

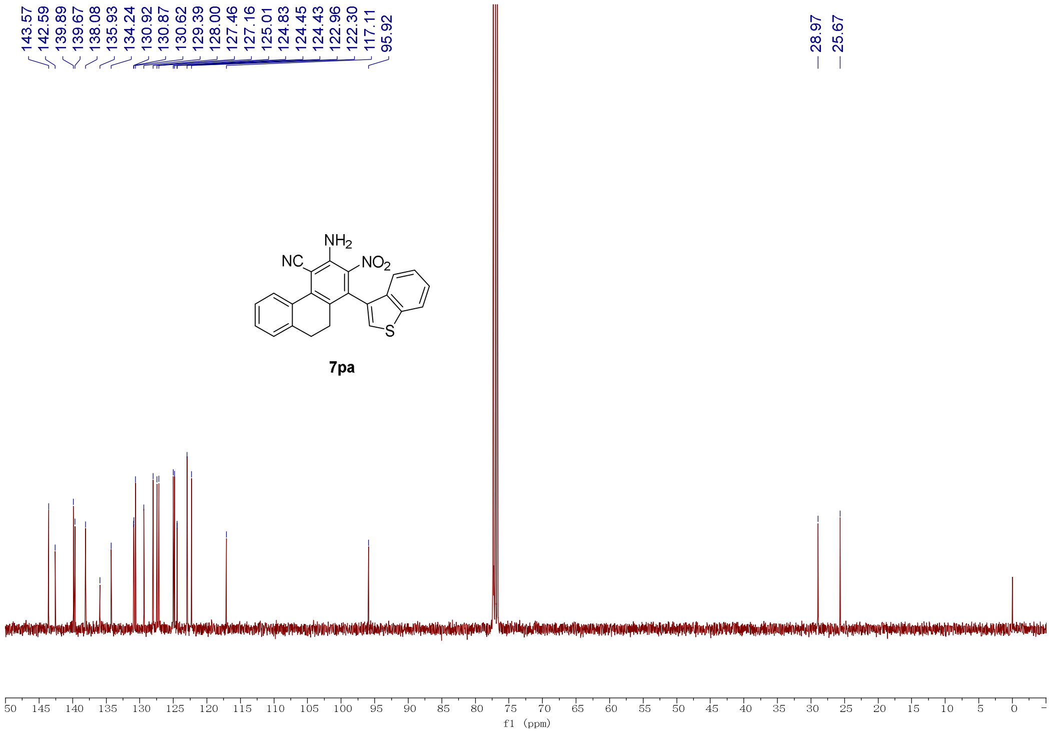
143.57
142.59
139.89
139.67
138.08
135.93
134.24
130.92
130.87
130.62
129.39
128.00
127.46
127.16
125.01
124.83
124.45
124.43
122.96
122.30
117.11
95.92
28.97
25.67
NH2
NC
NO2
S
7pa
f1 (ppm)

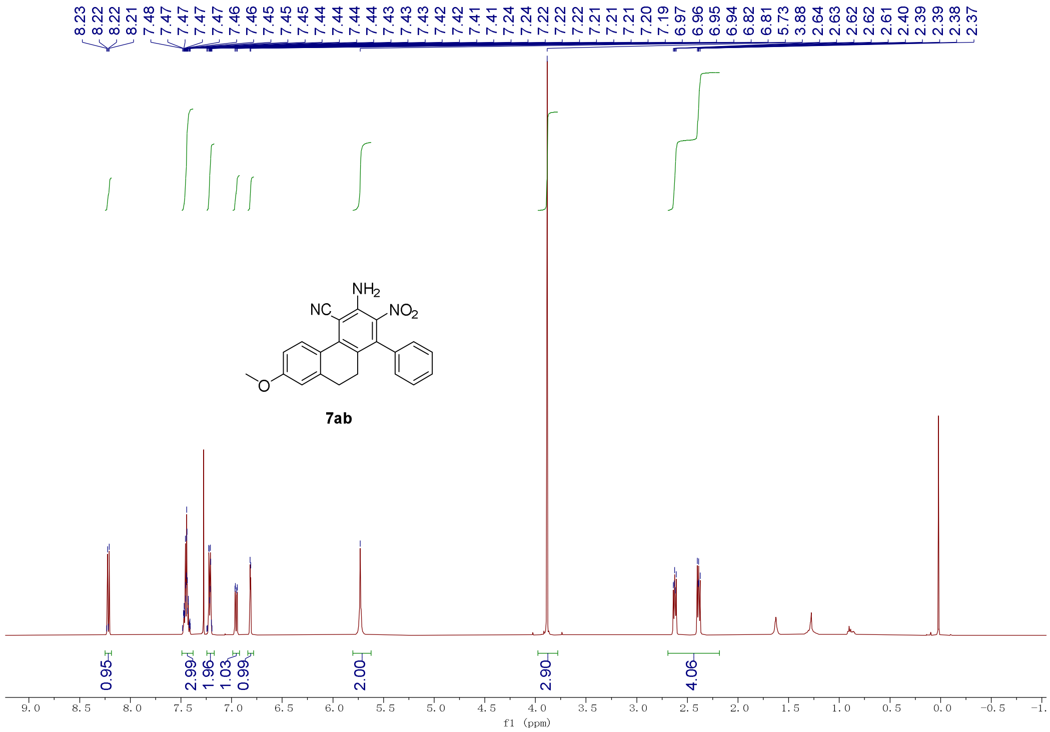

7ab

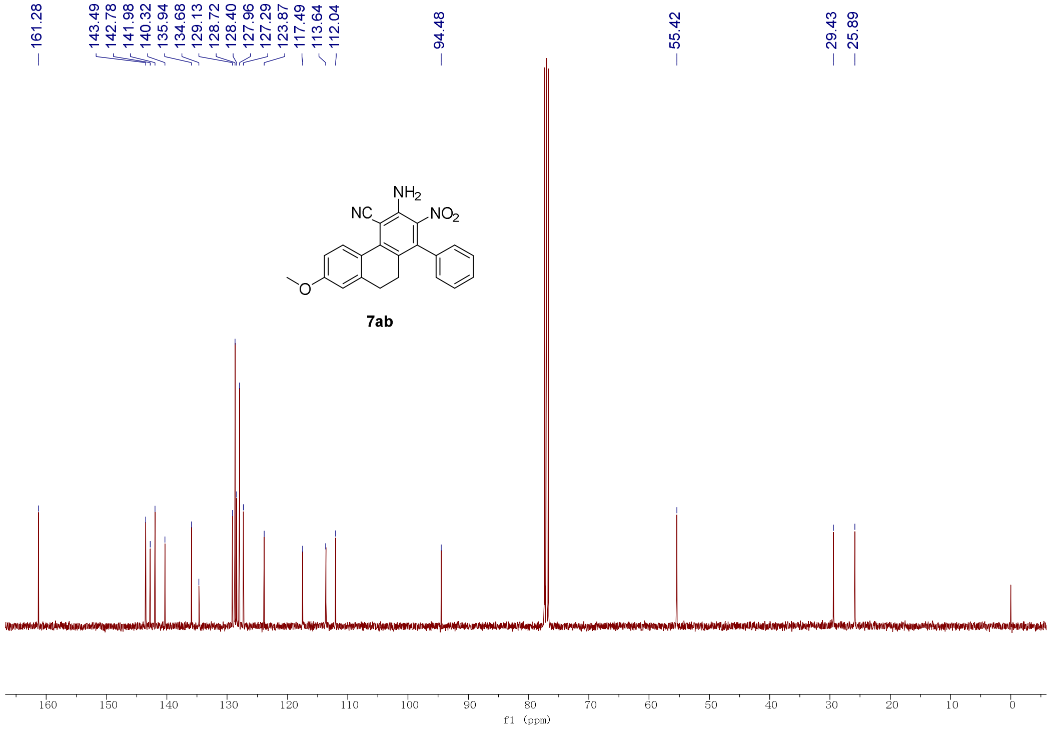

7ab

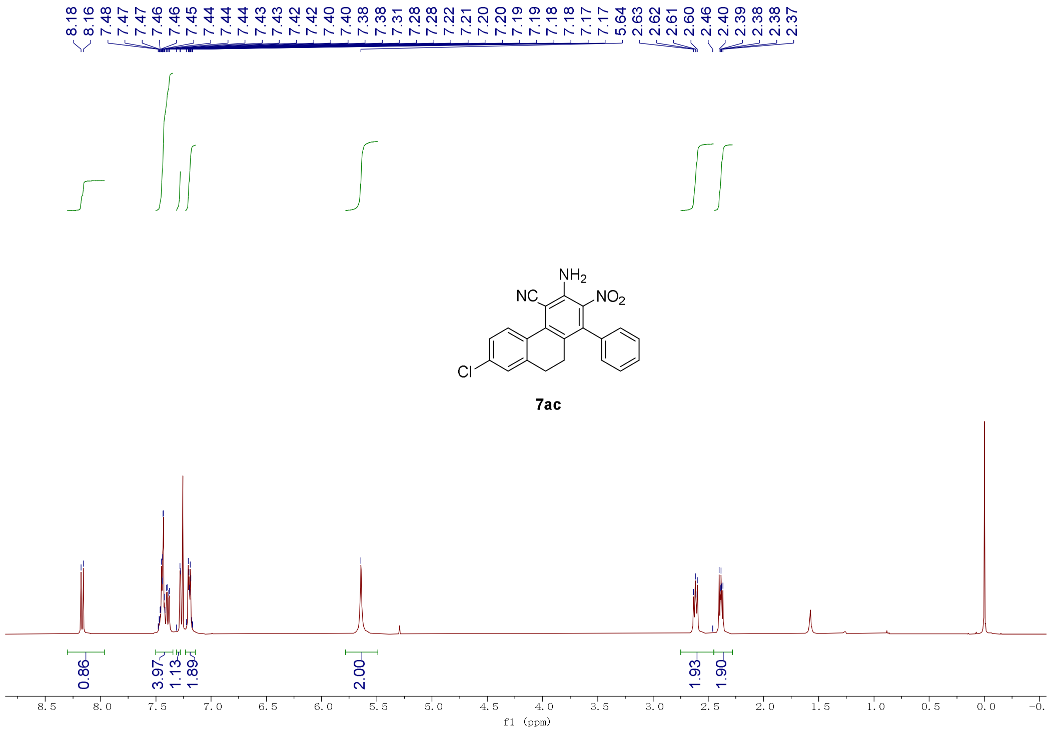

8.18
8.16
7.48
7.47
7.47
7.46
7.46
7.45
7.44
7.44
7.44
7.43
7.43
7.42
7.42
7.40
7.40
7.38
7.38
7.31
7.28
7.28
7.22
7.21
7.20
7.20
7.19
7.19
7.18
7.18
7.17
7.17
5.64
2.63
2.62
2.61
2.60
2.46
2.40
2.39
2.38
2.38
2.37
NH2
NC
NO2
Cl
7ac
0.86
3.97
1.13
1.89
2.00
1.93
1.90
f1 (ppm)

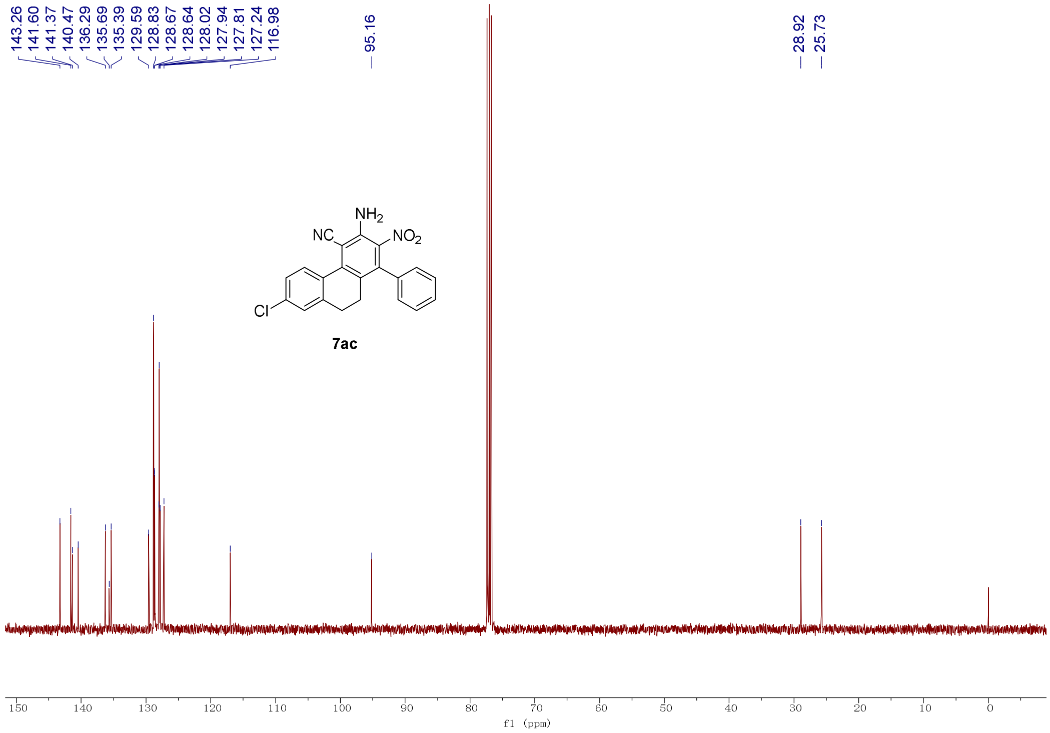

143.26
141.60
141.37
140.47
136.29
135.69
135.39
129.59
128.83
128.67
128.64
128.02
127.94
127.81
127.24
116.98
95.16
28.92
25.73
NH2
NC
NO2
Cl
7ac
f1 (ppm)

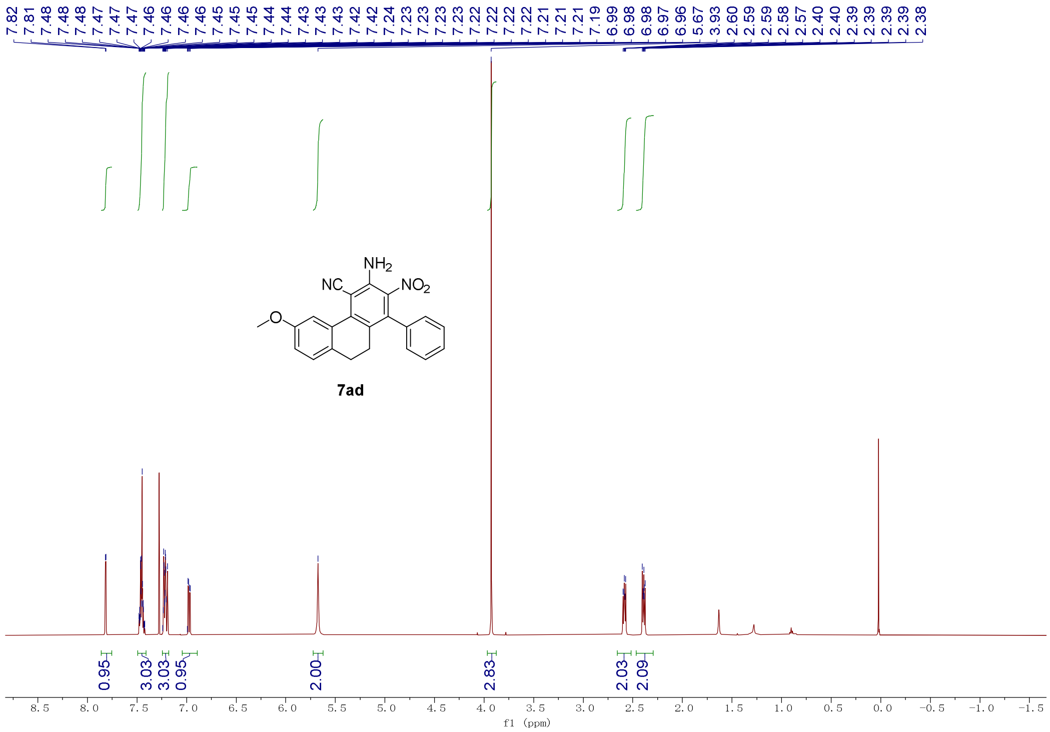
7.82 7.81 7.48 7.48 7.48 7.47 7.47 7.46 7.46 7.46 7.45 7.45 7.44 7.44 7.43 7.43 7.43 7.42 7.42 7.24 7.23 7.23 7.23 7.23 7.22 7.22 7.22 7.22 7.21 7.21 7.21 7.19 6.99 6.98 6.98 6.97 6.96 5.67 3.93 2.60 2.59 2.59 2.58 2.57 2.40 2.39 2.39 2.39 2.39 2.38
NH2
NC
NO2
O
7ad
0.95
3.03
3.03
0.95
2.00
2.83
2.03
2.09
f1 (ppm)

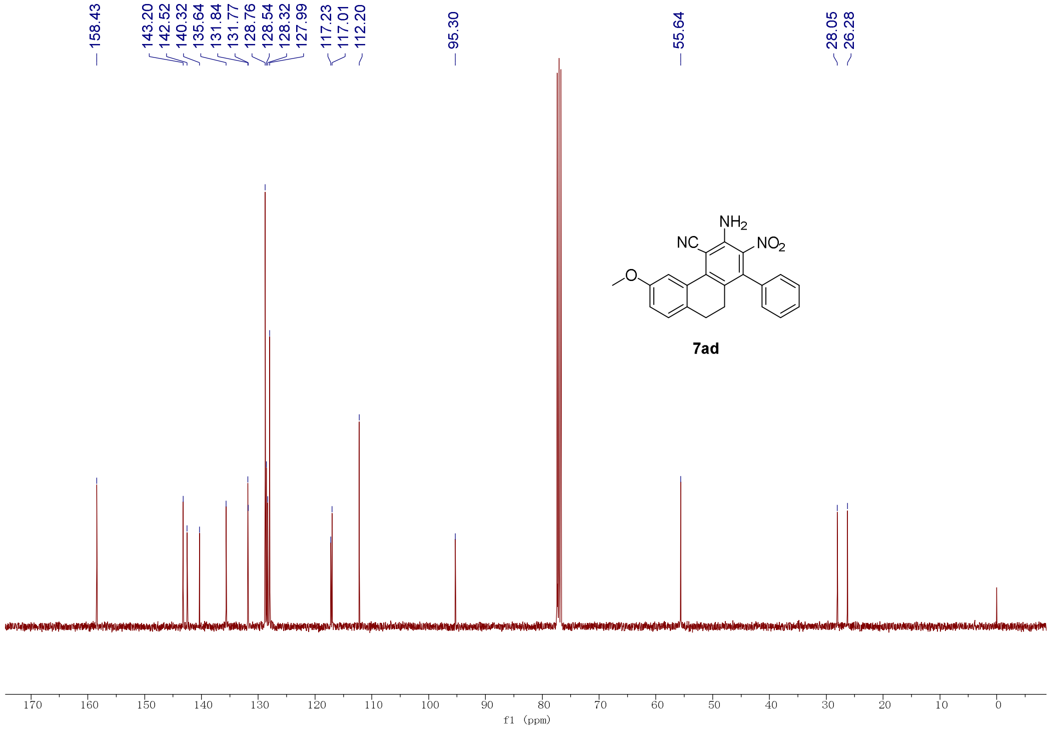
158.43
143.20 142.52 140.32 135.64 131.84 131.77 128.76 128.54 128.32 127.99
117.23 117.01 112.20
95.30
55.64
28.05 26.28
NH2
NC
NO2
O
7ad
f1 (ppm)

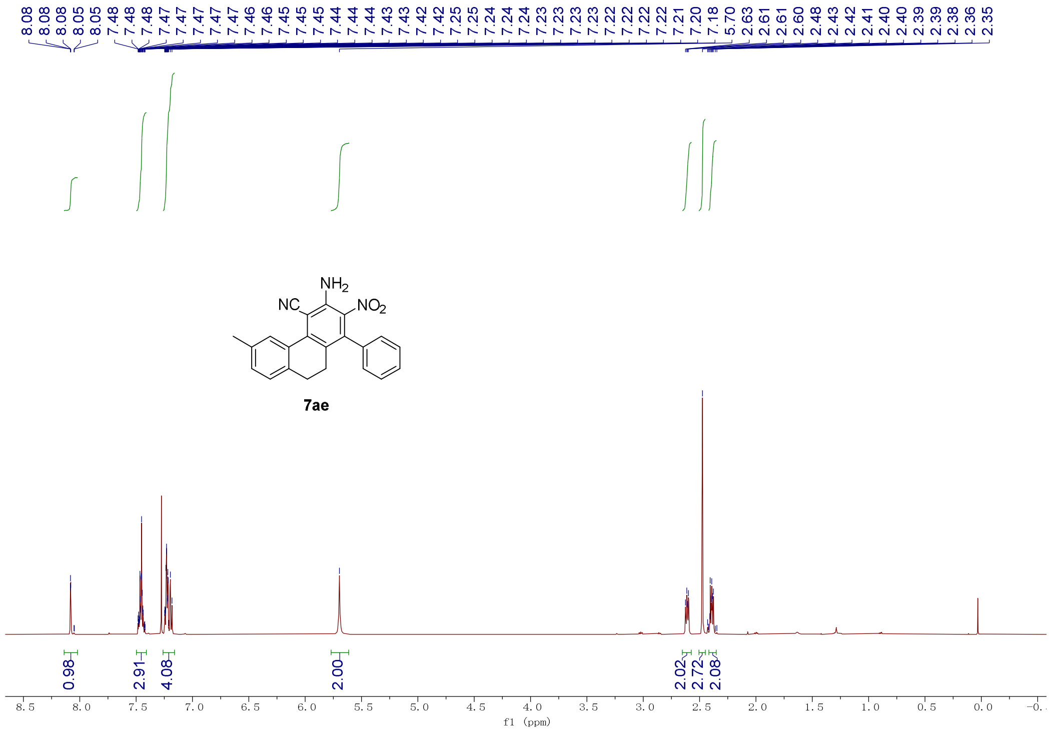
8.08 8.08 8.08 8.05 8.05 7.48 7.48 7.47 7.47 7.47 7.47 7.46 7.45 7.45 7.45 7.44 7.44 7.44 7.43 7.43 7.42 7.42 7.25 7.25 7.24 7.24 7.24 7.23 7.23 7.23 7.23 7.22 7.22 7.22 7.22 7.21 7.20 7.18 5.70 2.63 2.61 2.61 2.60 2.48 2.43 2.42 2.41 2.40 2.40 2.39 2.39 2.38 2.36 2.35
NH2
NC
NO2
7ae
0.98
2.91
4.08
2.00
2.02
2.72
2.08
f1 (ppm)

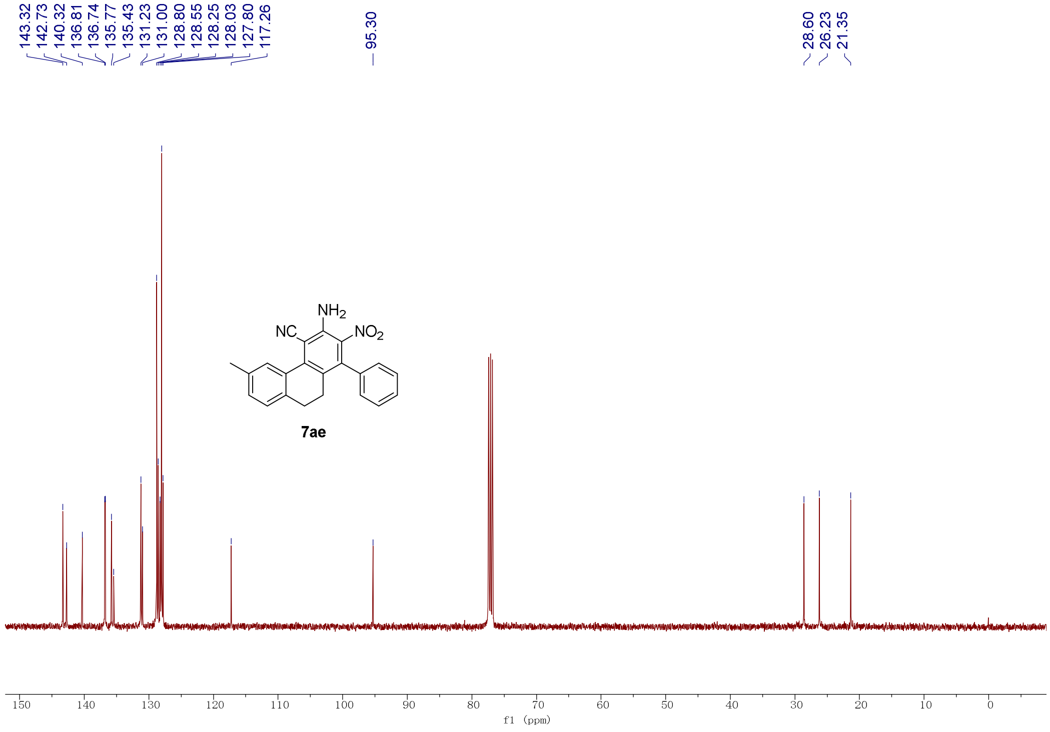
143.32 142.73 140.32 136.81 136.74 135.77 135.43 131.23 131.00 128.80 128.55 128.25 128.03 127.80 117.26
95.30
28.60 26.23 21.35
NH2
NC
NO2
7ae
f1 (ppm)

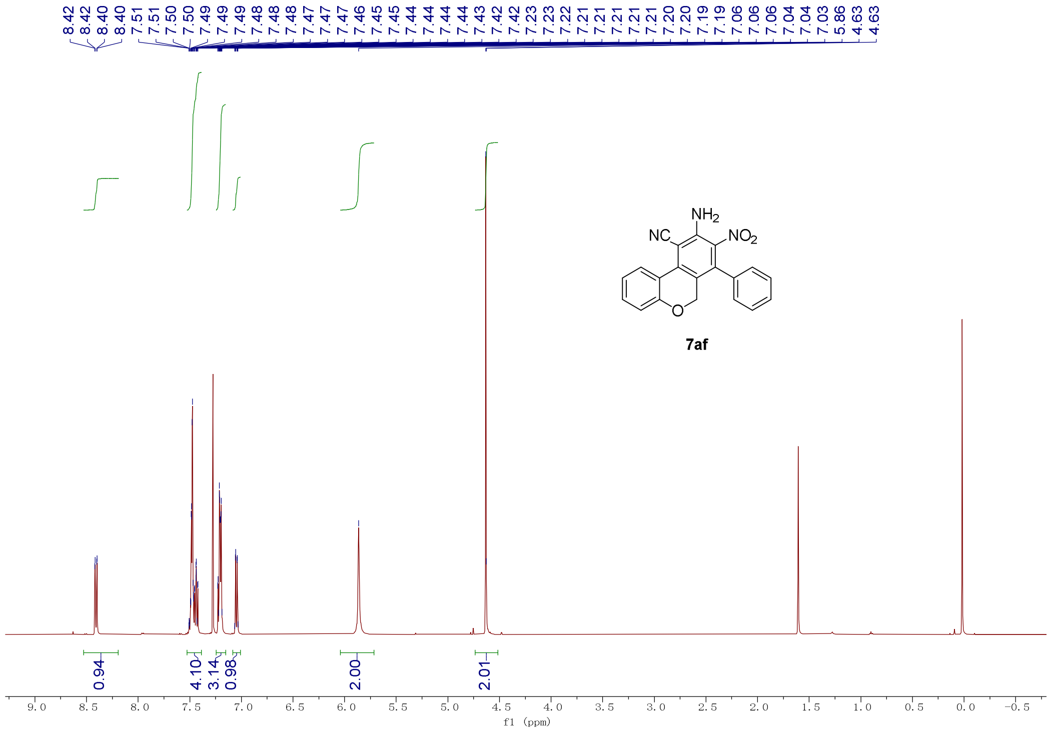

8.42
8.42
8.40
8.40
7.51
7.51
7.50
7.49
7.49
7.49
7.48
7.48
7.47
7.47
7.47
7.46
7.45
7.45
7.44
7.44
7.44
7.44
7.43
7.42
7.42
7.23
7.23
7.22
7.21
7.21
7.21
7.21
7.21
7.20
7.20
7.19
7.19
7.06
7.06
7.04
7.04
7.03
5.86
4.63
4.63
NH2
NC
NO2
O
7af
0.94
4.10
3.14
0.98
2.00
2.01
9.0
8.5
8.0
7.5
7.0
6.5
6.0
5.5
5.0
4.5
4.0
3.5
3.0
2.5
2.0
1.5
1.0
0.5
0.0
-0.5
f1 (ppm)

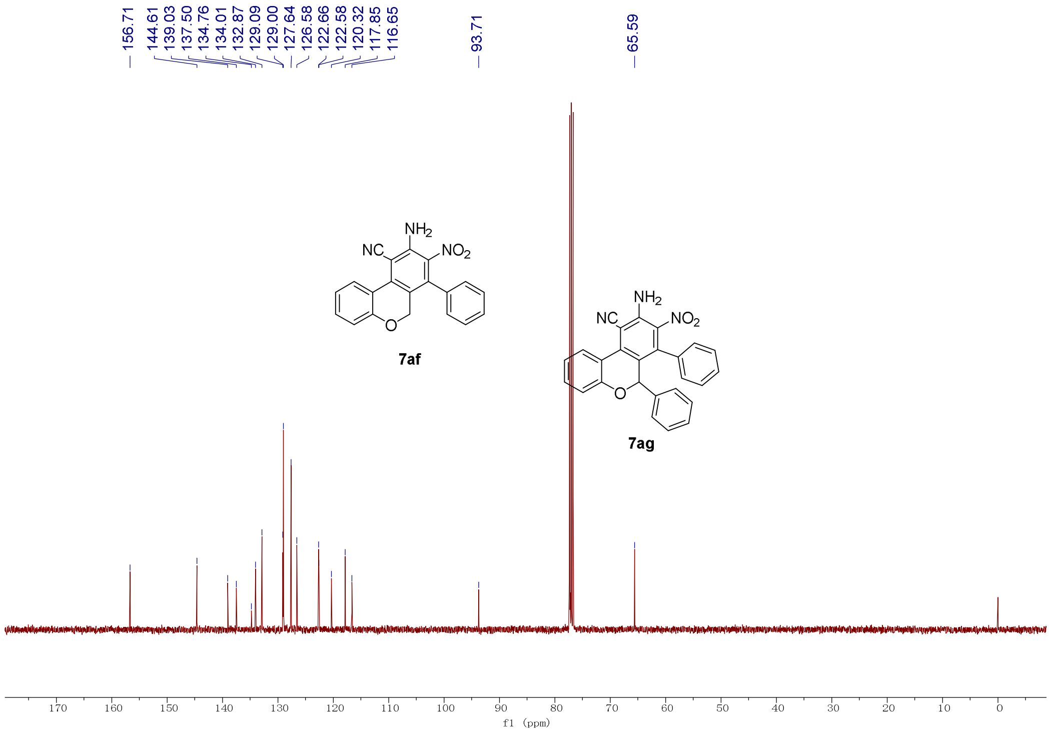
156.71
144.61
139.03
137.50
134.76
134.01
132.87
129.09
129.00
127.64
126.58
122.66
122.58
120.32
117.85
116.65
93.71
65.59
NH2
NC
NO2
O
7af
7ag
f1 (ppm)

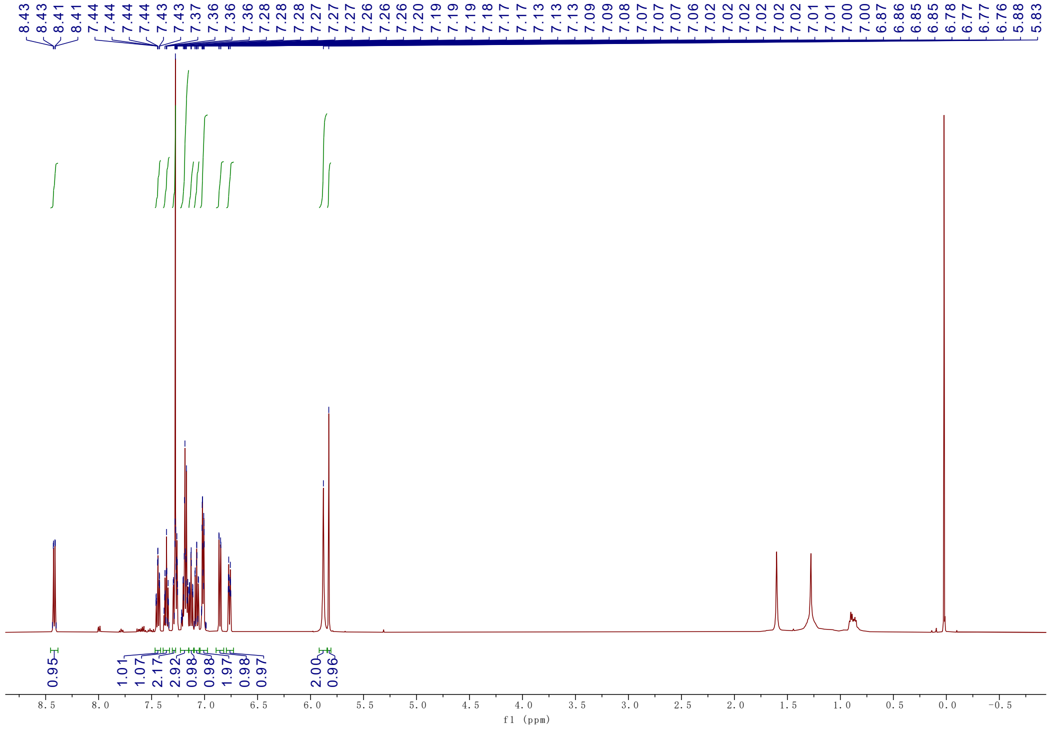
8.43
8.43
8.41
8.41
7.44
7.44
7.44
7.43
7.43
7.37
7.36
7.36
7.28
7.28
7.28
7.27
7.27
7.27
7.26
7.26
7.20
7.19
7.19
7.19
7.18
7.17
7.17
7.13
7.13
7.13
7.09
7.08
7.07
7.07
7.07
7.06
7.02
7.02
7.02
7.02
7.02
7.02
7.01
7.01
7.00
7.00
6.87
6.86
6.85
6.85
6.78
6.77
6.77
6.76
5.88
5.83
0.95
1.01
1.07
2.17
2.92
0.98
0.98
1.97
0.98
0.97
2.00
0.96
f1 (ppm)

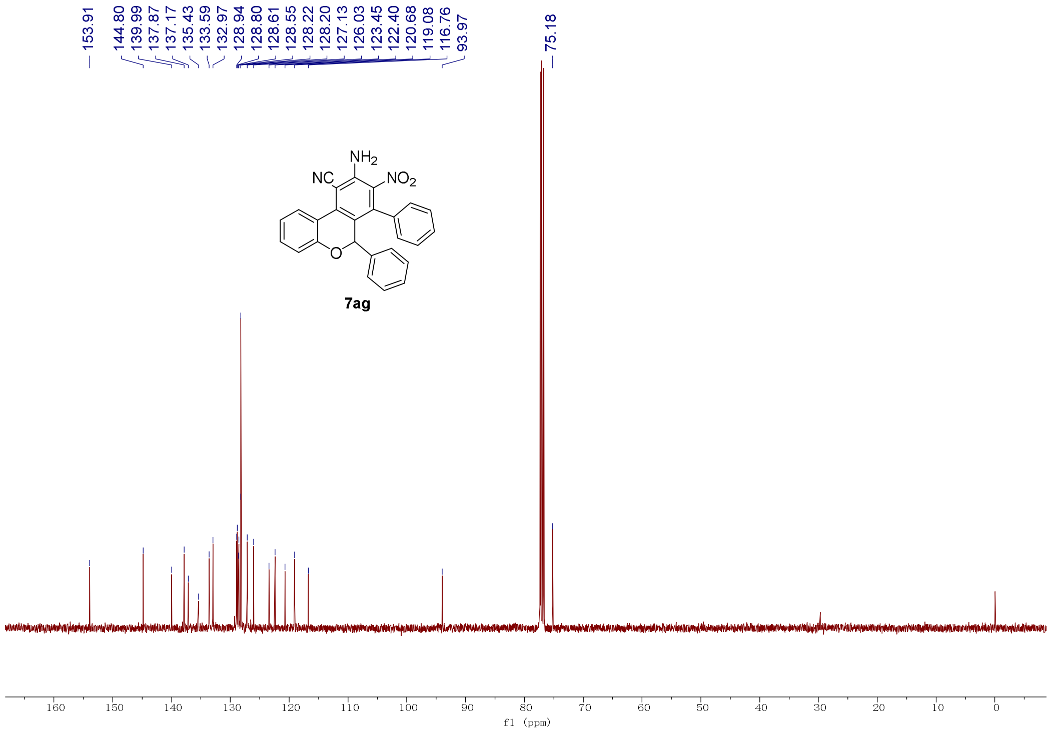
153.91
144.80
139.99
137.87
137.17
135.43
133.59
132.97
128.94
128.80
128.61
128.55
128.22
128.20
127.13
126.03
123.45
122.40
120.68
119.08
116.76
93.97
75.18
NH2
NC
NO2
O
7ag
160
150
140
130
120
110
100
90
80
70
60
50
40
30
20
10
0
f1 (ppm)

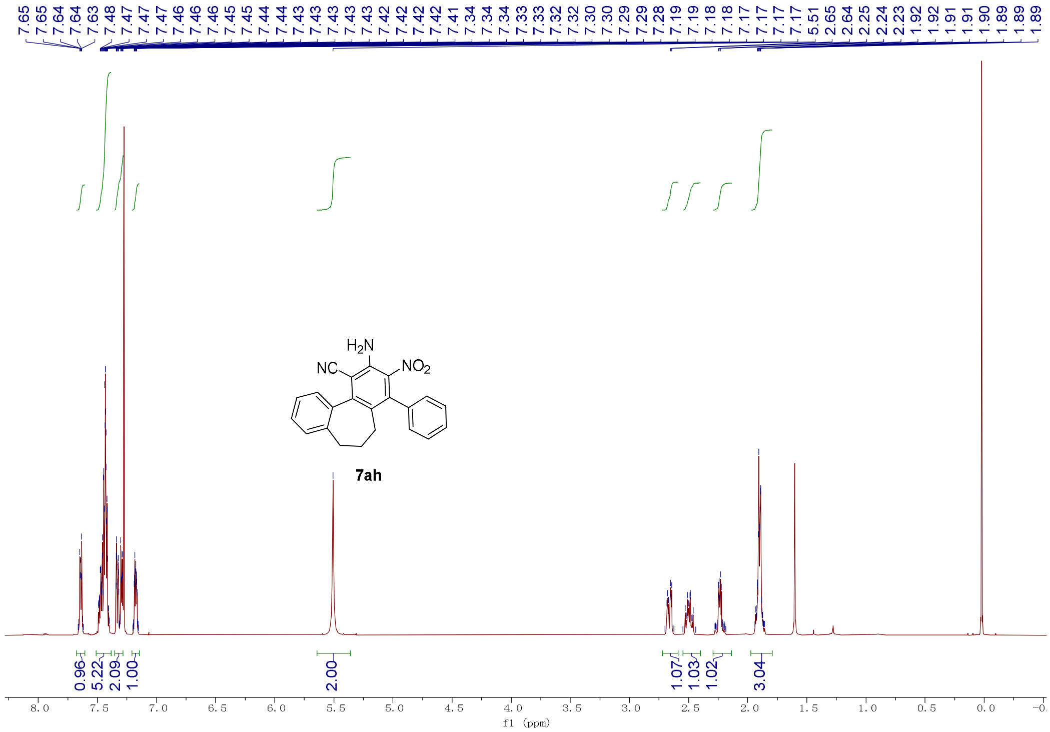
7ah
f1 (ppm)

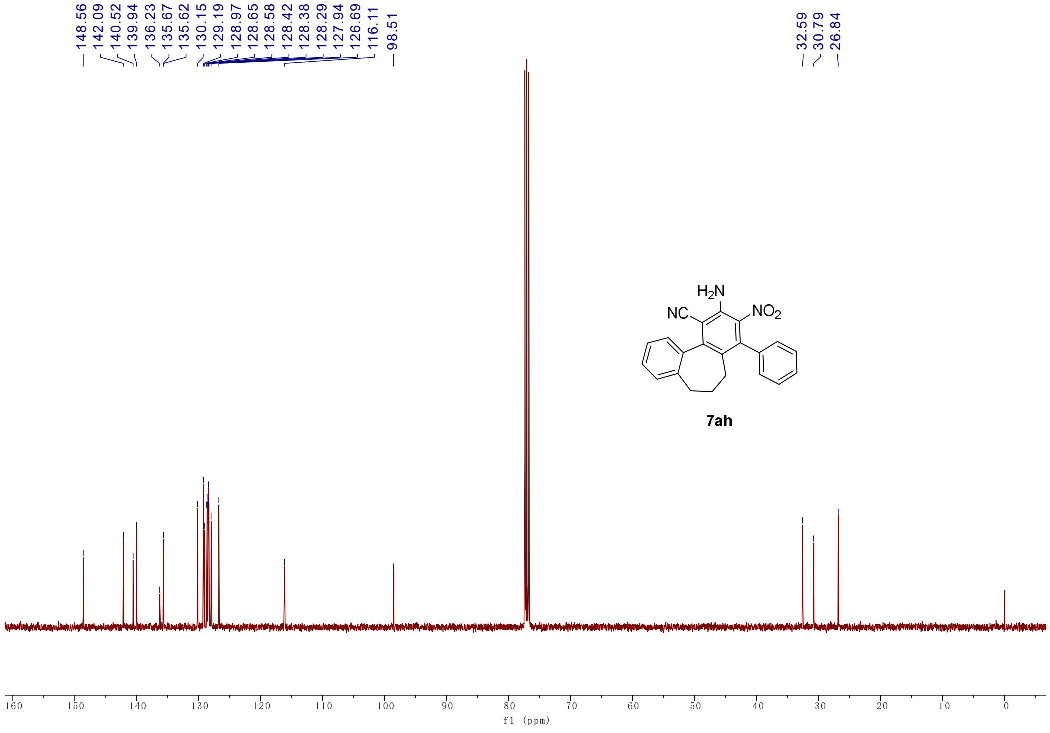
7ah
f1 (ppm)

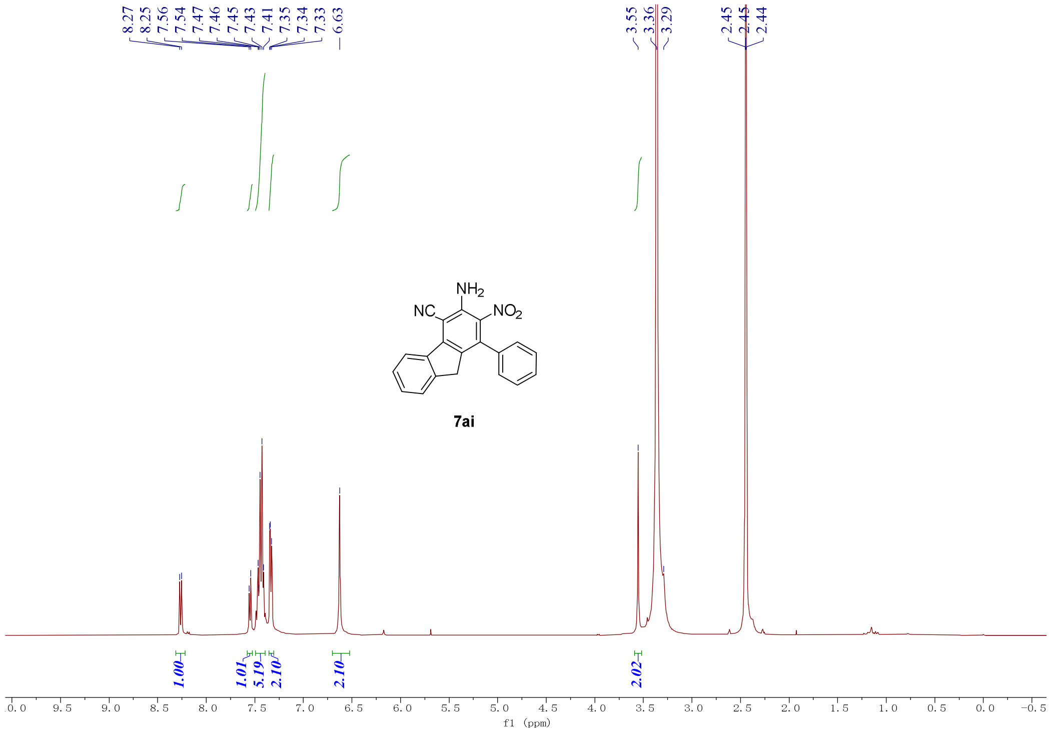

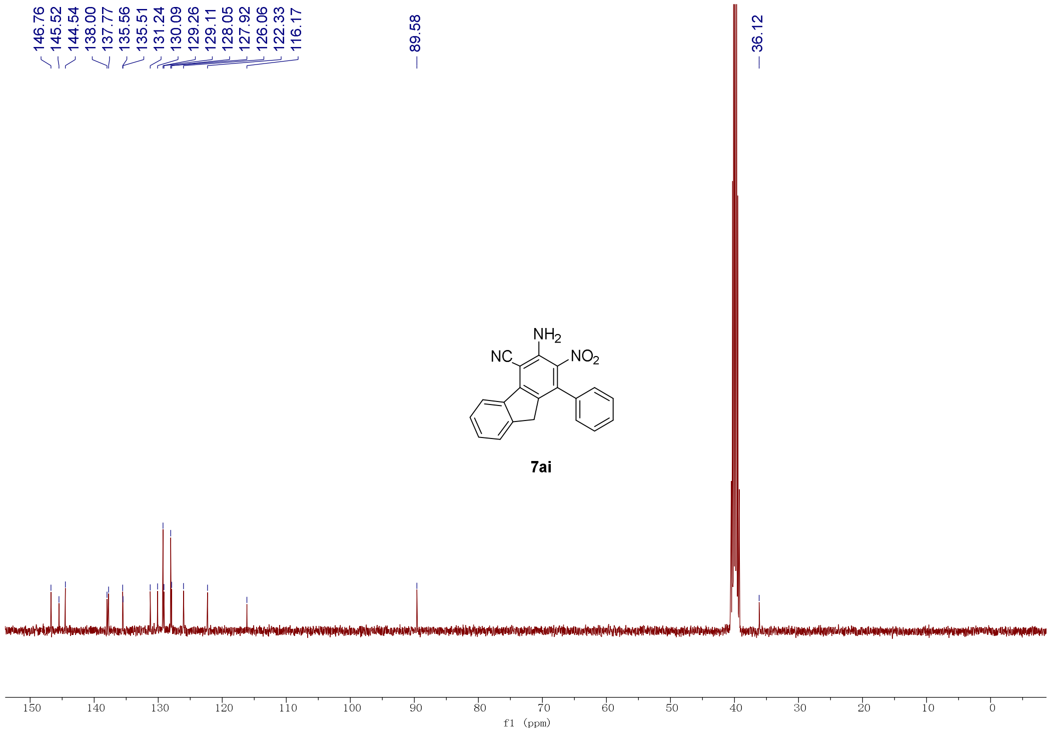

4. Reference

1. Pan, H.; Han, M.-Y.; Li, P.; Wang, L. "On Water" Direct Catalytic Vinylogous Aldol Reaction of Silyl Glyoxylates. *J. Org. Chem.* **2019**, *8421*, 14281-14290.
2. Bauvois, B.; Puiffe, M.-L.; Bongui, J.-B.; Paillat, S.; Monneret, C.; Dauzonne, D. Synthesis and biological evaluation of novel flavone-8-acetic acid derivatives as reversible inhibitors of aminopeptidase N/CD13. *J. Med. Chem.* **2003**, *46*, 3900–3913.
3. Yang, W.; Du, D.-M. Highly enantioselective Michael addition of nitroalkanes to chalcones using chiral squaramides as hydrogen bonding organocatalysts. *Org. Lett.* **2010**, *12*, 5450-5453.
4. Requena, J. V.A.; Lopez, E.M.; Herrera, R. P. One-pot synthesis of unsymmetrical squaramides. *RSC Adv.* **2015**, *5*, 33450-33462.
